# Correction: Prevalence of mental health problems among children with long COVID: A systematic review and meta-analysis

**DOI:** 10.1371/journal.pone.0296160

**Published:** 2023-12-14

**Authors:** The PLOS ONE Staff

## Notice of Republication

This article was republished on December 7, 2023, to correct errors in the figures that were introduced during the typesetting process. The publisher apologizes for the errors. Please download this article again to view the correct version. The originally published, uncorrected article and the republished, corrected articles are provided here for reference.

## Supporting information

S1 File Originally published, uncorrected article. (PDF)

S2 File Republished, corrected article. (PDF)

## References

[1] Mat Hassan N, Salim HS, Amaran S, Yunus NI, Yusof NA, Daud N, Fry D. (2023) Prevalence of mental health problems among children with long COVID: A systematic review and meta-analysis. PloS ONE 18(5): e0282538. doi: 10.1371/journal.pone.0282538 37195978 PMC10191312

