# Supplementary material for: Prevalence of mental health problems among children with long COVID: A systematic review and meta-analysis
Source: PLoS One. 2023 May 17;18(5):e0282538. doi: 10.1371/journal.pone.0282538 (PMC10191312; doi:10.1371/journal.pone.0282538)
Supplement: S1 Table — (DOCX) [file pone.0282538.s001.docx]

**S1 Table**: Summary of studies assessing each mental health outcomes with pooled prevalence results

| Mental health outcomes | Studies (First author, year) | Assessment type | No of samples | Population  Age  %males | Assessment times post infection | Proportion with outcome  (95% CI) | Pooled prevalence (95% CI) | Heterogeneity  I^2^ | *p*-value |
| --- | --- | --- | --- | --- | --- | --- | --- | --- | --- |
| Anxiety | Zhang 2021 | Screen for Child Anxiety Related Emotional Disorders (SCARED) | 1257 | Hospital  15.8% adolescents  64.5% males | Four to six months | 23%  (13, 35) | 9% (1, 23) | 84% | *p*<0.01 |
|  | Zavala 2021 | Presence of symptom assessing frequency | 859 | Community  6-13 years old  Median 10  51.2% | Minimum one month | 7%  (5, 10) |  |  |  |
|  | Clavenna 2022 | Checklist for symptoms with yes/no answers | 148 | Outpatient  0-16 years old  Median age: 6.5 (IQR:3.5-10.5)  50% | 6 months | 2%  (0.01, 13) |  |  |  |
| Depression | Zhang 2021 | 10-item Children's Depression Inventory-Short version (CDI-S) | 1257 | Hospital  15.8% adolescents  64.5% males | Four to six months | 48%  (35, 61) | 15% (0.04, 0.47) | 97% | *p*<0.01 |
|  | Zavala 2021 | Presence of symptoms assessing frequency | 859 | Community  6-13 years old  Median 10  51.2% | Minimum one month | 4%  (2, 6) |  |  |  |
|  | Sterky 2021 | Single symptom yes/no question | 55 | Hospital  0-18 years old  58% | Four to ten months | 5.5%  (1, 15) |  |  |  |
| Concentration difficulties | Berg  2022 | Single symptom questions. Assessed presence of symptom before and after | 28270  (6630 cases, 21640 controls) | Community  15-18 years old  Median 17.6  42.4% | Two months to 17 months | 12%  (11, 13) | 6% (3, 11) | 99% | *p*<0.01 |
|  | Borch 2022 | Single symptom yes/no question | 15041 cases  15080 controls | Community  0 – 17 years old | One months to 14 months | 6%  (6, 7) |  |  |  |
|  | Molteni 2021 | Single symptom yes/no question | 1734 cases  1734 controls | Community, online  5-17 years old | Day 28 until end of illness | 0.3%.  (0.1, 1) |  |  |  |
|  | Roge 2021 | Post-COVID-19 symptom assessment | 378 | Community  1 month to 18 years old. Median age: 6.5 (IQR:3.5-10.5) | One to 6 months post infection | 17%  (12, 22) |  |  |  |
|  | Stephenson 2022 | Symptoms assessed as present or absent | 3065 cases  3739 controls | Community  11- 17 years old | 3 months | 6%  (6, 7) |  |  |  |
|  | Sterky 2021 | Single symptom questions. | 55  No controls | Hospital  0-18 years old  58% | Four to 10 months post discharge | 5%  (1, 15) |  |  |  |
|  | Zavala 2021 | Presence of symptoms assessing frequency | 859 | Community  6-13 y.o  Median 10  51.2% | Minimum one month | 4%  (2, 6) |  |  |  |
| Sleep disturbances | Zhang 2021 | 26-item Sleep Disturbance Scale for Children (SDSC)  Others | 1257 | Hospital  15.8% adolescents  64.5%males | Four to six months | 6%  (6, 7) | 9% (5, 13) | 79% | *p*<0.01 |
|  | Buonsenso 2021 | Single symptom questions. | 129 | Clinic patients | One month | 19% (12, 26) |  |  |  |
|  | Osmanov 2021 | Single symptom yes/no question | 518 | Hospital  Median age 10.4  46.9% | 7-9 months | 7% (5, 9) |  |  |  |
|  | Clavenna 2022 | Checklist for symptoms with yes/no answers. | 148  2% | Outpatient paeds  0-16  Median age: 6.5 (IQR:3.5-10.5) | 6 months | 10% (2, 23) |  |  |  |
|  | Zavala 2021 | Presence of symptoms assessing frequency | 859 | Community  6-13 y.o  Median 10  51.2% | Minimum one month | 7%  (5, 10) |  |  |  |
| Mood swings | Berg 2022 | Presence of symptoms assessing frequency | 28270  (6630 cases, 21640 controls)859 | Community  6-13 y.o  Median 10  51.2% | Minimum one month | 11%  (10, 12) | 13% (5, 23) | 95% | *p*<0.01 |
|  | Roge 2021 | Post-COVID-19 symptom assessment | 378 | Community  1 month to 18 years old | One to 6 months post infection | 23%  (18, 29) |  |  |  |
|  | Zavala 2021 | Presence of symptoms assessing frequency | 859 | Community  6-13 y.o  Median 10  51.2% | Minimum one month | 7%  (5, 9) |  |  |  |
| Appetite  problems | Berg 2022 | Presence of symptoms assessing frequency | 28270  (6630 cases, 21640 controls) | Community  6-13 y.o  Median 10  51.2% | Minimum one month | 8%  (7, 9) | 5% (1, 13) | 99% | *p*<0.01 |
|  | Molteni 2021 | Single symptom yes/no question | 1734 cases 1734 controls | Community, online  5-17 years old | Day 28 until end of illness | 1%  (0.1, 1) |  |  |  |
|  | Stephenson 2022 | Symptoms assessed as present or absent | 3065 cases 3739 | Community | 3 months | 10% (9, 11) |  |  |  |
|  |  |  |  |  |  |  |  |  |  |
