# Supplementary material for: Prevalence of mental health problems among children with long COVID: A systematic review and meta-analysis
Source: PLoS One. 2023 May 17;18(5):e0282538. doi: 10.1371/journal.pone.0282538 (PMC10191312; doi:10.1371/journal.pone.0282538)
Supplement: S2 Table — (DOCX) [file pone.0282538.s002.docx]

## S2 Table: Example of Risk of Bias/ Quality Assessment

| NIH | Quality Assessment | | | |
| --- | --- | --- | --- | --- |
|  | 1.Blackenburgh |  | 2.Berg |  |
| Observational cohort/ Cross-sectional | R1 | R2 | R1 | R2 |
| 1. Was the research question or objective in this paper clearly stated? | Y | Y | Y | Y |
| 2. Was the study population clearly specified and defined? | Y | Y | Y | Y |
| 3. Was the participation rate of eligible persons at least 50%? | Y | Y | N | N |
| 4. Were all the subjects selected or recruited from the same or similar populations (including the same time period)? | Y | Y | Y | Y |
| Were inclusion and exclusion criteria for being in the study prespecified and applied uniformly to all participants? | Y | Y | Y | Y |
| 5. Was a sample size justification, power description, or variance and effect estimates provided? | N | N | Y | Y |
| 6. For the analyses in this paper, were the exposure(s) of interest measured prior to the outcome(s) being measured? | N | N | Y | Y |
| 7. Was the timeframe sufficient so that one could reasonably expect to see an association between exposure and outcome if it existed? | N | N | Y | Y |
| 8. For exposures that can vary in amount or level, did the study examine different levels of the exposure as related to the outcome | Y | Y | Y | Y |
| 9. Were the exposure measures (independent variables) clearly defined, valid, reliable, and implemented consistently across all study participants? | N | N | Y | Y |
| 10. Was the exposure(s) assessed more than once over time? | Y | Y | N | N |
| 11. Were the outcome measures (dependent variables) clearly defined, valid, reliable, and implemented consistently across all study participants? | Y | Y | Y | Y |
| 12. Were the outcome assessors blinded to the exposure status of participants? | N | N | N | N |
| 13. Was loss to follow-up after baseline 20% or less? | N | N | NA | NA |
| 14. Were key potential confounding variables measured and adjusted statistically for their impact on the relationship between exposure(s) and outcome(s)? | N | N | Y | Y |
| Quality (/) Comments |  |  |  |  |
| Good |  |  | / | / |
| Moderate |  |  |  |  |
| Low | / | / |  |  |
| Included (Y/N) | N | N | Y | Y |
