# Supplementary figures and images for: Prevalence of mental health problems among children with long COVID: A systematic review and meta-analysis
Source: PLoS One. 2023 May 17;18(5):e0282538. doi: 10.1371/journal.pone.0282538 (PMC10191312; doi:10.1371/journal.pone.0282538)

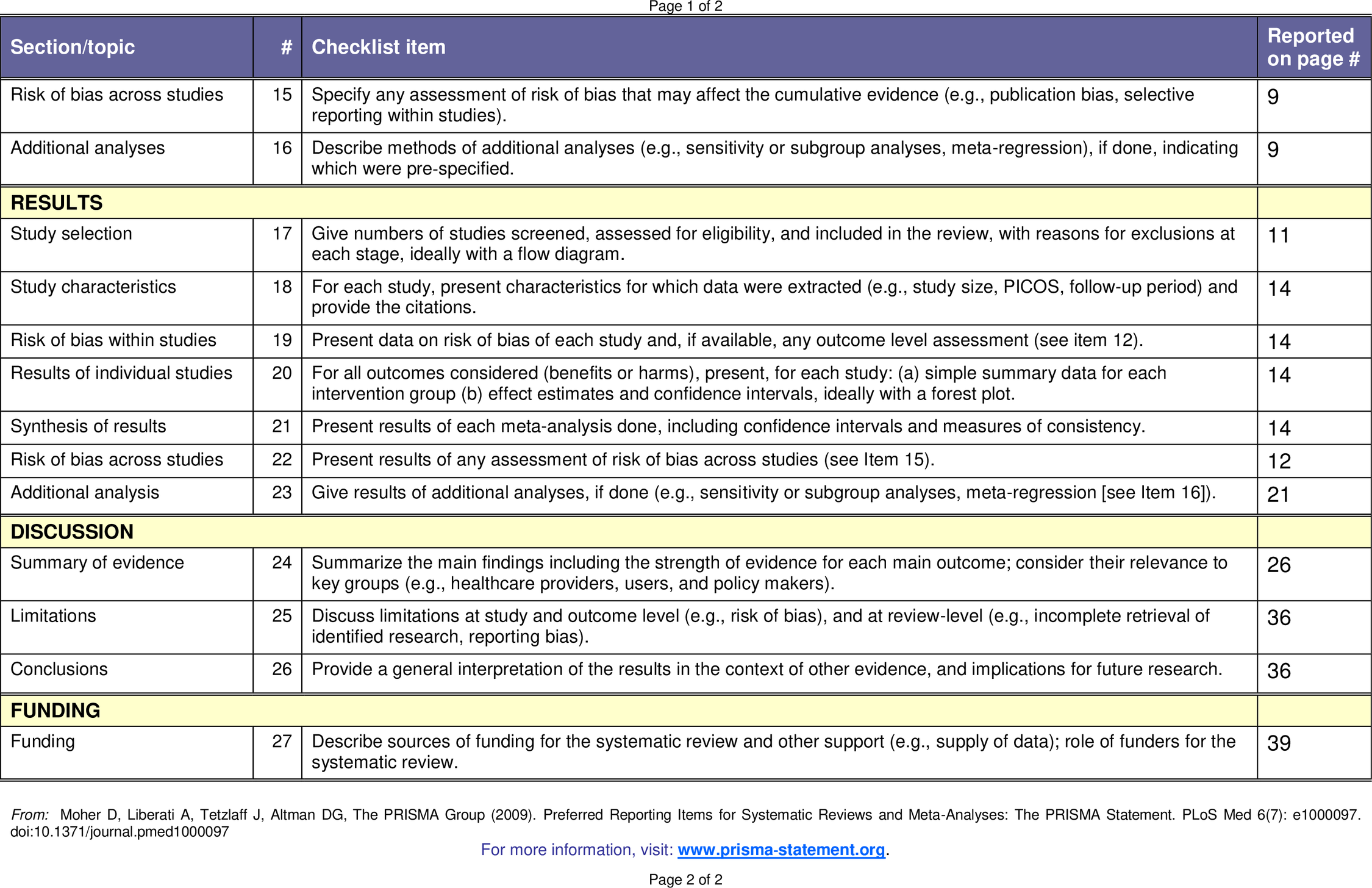

Supplement: S1 Checklist — (TIF) [file pone.0282538.s003.tif]
