# Supplementary material for: Correction: Prevalence of mental health problems among children with long COVID: A systematic review and meta-analysis
Source: PLoS One. 2023 Dec 14;18(12):e0296160. doi: 10.1371/journal.pone.0296160 (PMC10721052; doi:10.1371/journal.pone.0296160)
Supplement: S1 File — (PDF) [file pone.0296160.s001.pdf]

PLOS ONE

RESEARCH ARTICLE

Prevalence of mental health problems among children with long COVID: A systematic review and meta-analysis

Nurulhuda Mat Hassan[1,2]*, **Hani Syahida Salim**[3], **Safiya Amaran**[2], **Nurul Izza Yunus**[2], **Nurul Azreen Yusof**[2], **Norwati Daud**[2], **Deborah Fry**[1]*

1 University of Edinburgh, Edinburgh, Scotland, United Kingdom, **2** Faculty of Medicine, Universiti Sultan Zainal Abidin, Kuala Terengganu, Terengganu, Malaysia, **3** Faculty of Medicine, Universiti Putra Malaysia, Serdang, Selangor, Malaysia

* nurulhudamh@unisza.edu.my (NMH); debi.fry@ed.ac.uk (DF)

Check for updates

OPEN ACCESS

Citation: Mat Hassan N, Salim HS, Amaran S, Yunus NI, Yusof NA, Daud N, et al. (2023) Prevalence of mental health problems among children with long COVID: A systematic review and meta-analysis. PLoS ONE 18(5): e0282538. https://doi.org/10.1371/journal.pone.0282538

Editor: Omar Enzo Santangelo, Regional Health Care and Social Agency of Lodi, ITALY

Received: September 29, 2022

Accepted: February 16, 2023

Published: May 17, 2023

Copyright: © 2023 Hassan et al. This is an open access article distributed under the terms of the Creative Commons Attribution License, which permits unrestricted use, distribution, and reproduction in any medium, provided the original author and source are credited.

Data Availability Statement: All relevant data are within the manuscript and its Supporting Information files.

Funding: The author(s) received no specific funding for this work.

Competing interests: The authors have declared that no competing interests exist.

Abstract

Introduction

The number of children with mental health problems has more than doubled since the COVID-19 pandemic. However, the effect of long Covid on children's mental health is still debatable. Recognising long Covid as a risk factor for mental health problems in children will increase awareness and screening for mental health problems following COVID-19 infection, resulting in earlier intervention and lower morbidity. Therefore, this study aimed to determine the proportion of mental health problems post-COVID-19 infection in children and adolescents, and to compare them with the population with no previous COVID-19 infection.

Methodology

A systematic search was done in seven databases using pre-defined search terms. Cross-sectional, cohort and interventional studies reporting the proportion of mental health problems among children with long COVID in the English language from 2019 to May 2022 were included. Selection of papers, extraction of data and quality assessment were done independently by two reviewers. Studies with satisfactory quality were included in meta-analysis using R and Revman software programmes.

Results

The initial search retrieved 1848 studies. After screening, 13 studies were included in the quality assessments. Meta-analysis showed children who had previous COVID-19 infection had more than two times higher odds of having anxiety or depression, and 14% higher odds of having appetite problems, compared to children with no previous infection. The pooled prevalence of mental health problems among the population were as follows; anxiety: 9% (95% CI:1, 23), depression: 15%(95% CI:0.4, 47), concentration problems: 6%(95% CI: 3, 11), sleep problems: 9%(95% CI:5, 13), mood swings: 13% (95%CI:5, 23) and appetite loss: 5%(95% CI:1, 13). However, studies were heterogenous and lack data from low- and middle-income countries.

Conclusion

Anxiety, depression and appetite problems were significantly increased among post-COVID-19 infected children, compared to those without a previous infection, which may be attributed to long COVID. The findings underscore the importance of screening and early intervention of children post-COVID-19 infection at one month and between three to four months.

Introduction

As the world is reeling from the impact of the COVID-19 pandemic, the population recovering from COVID-19 infection grows. This accounts for more than 600 million cases worldwide, and counting [1]. As the impact of the infection towards infected individuals involve mental health, this condition could potentially further exacerbate the problem of widened treatment gap in mental health problems, which was a focus of the global mental health agenda [2], or be an opportunity to build back the mental healthcare system, better. Evidence-based knowledge areas are crucial regarding mental health problems in long COVID among children, and would guide the policy-making and clinical decisions of healthcare professionals in the area.

The COVID-19 pandemic brought multiple negative impact transcending societies, socio-economic status and health sectors. Caused by the SARS-CoV-2 initially detected among humans in December 2019 in Wuhan, China [3], the infection had a rapid spread across nations and continents, leading the World Health Organization to announce it as a pandemic in March 2020 [4]. The pandemic then occurred in waves with many deadly variants such as Delta and Omicron being subsequently detected, leading to pandemic restrictions such as school closures, mandatory quarantine and travel restrictions being imposed. Post-COVID-19 vaccine implementation saw the pandemic restrictions being relaxed and slowly abolished, but despite a 'near-normal' regulations, the world is still reeling from the trans-sectoral impact of the pandemic, including in the area of mental health.

Mental health problems such as anxiety and depression were shown to be increased substantially post-pandemic. Recent data specifically for children and adolescents found the global pooled prevalence of depression and anxiety to be 25.2% and 20.5% respectively [5]. These were significant increases compared to a global prevalence estimated in 2015, where the estimated prevalence were 6.5% for anxiety disorders and 2.6% for any depressive disorders [6]. Data from the United States in 2017 was higher with 12.9% prevalence of depression among 12 to 17 years old children [7]. These data showed a major post-pandemic increase, which have at least doubled the prevalence of depression and anxiety symptoms pre-COVID-19. These increases were a collective effect of the pandemic, whether due to the SARS-CoV-2 infection itself or as an impact from the social restrictions and other effects of the pandemic.

Until recently, studies investigating the direct effect of COVID-19 mainly focused on adults. The studies investigating mental health problems were relatively less, and there were even fewer studies conducted which focused especially on mental health problems in long COVID among children. Thus, it is difficult to estimate how far mental health problems in children have intensified due to the SARS-CoV-2 infection itself. This has impacted the recognition of this problem in long COVID and thus makes it hard for parents and children to access the screening and intervention they need. Indeed, earlier in the pandemic, parents struggled to obtain support for their children with long COVID and they felt dismissed when consulting their doctors [8]. This issue also impacts the policies of healthcare providers and the allocations

of funds to tackle the problem. It is also a hindrance in the efforts of global mental health movements to ensure an increase in access and screening for mental health problems, making it a global issue to solve. Up to date, there is yet any confirmatory studies that investigate the mental health effect of long COVID in children with previous COVID-19 infection.

Despite primarily infecting the respiratory system, SARS-CoV-2 infection may also involve multiple organs. The impact of this infection can be prolonged beyond four weeks in 20% of people, manifesting as physical and psychological symptoms [9], which is termed as long COVID. This prolonged condition, characterized as ongoing symptomatic COVID-19, is also known by many other terms: post-COVID conditions, post-acute COVID-19, post-acute COVID syndrome, chronic COVID, long-haul COVID and post-acute sequalae of SARS--COV-2 infection (PASC) [10]. Studies have shown that 10% of patients even have the symptoms persist after 12 weeks, classified as post-COVID-19 syndrome [11]. Up to date, there is still some debate regarding the definition of long COVID, as WHO has defined it to be more than eight weeks [12], as opposed to the more than four weeks classification by the National Institute for Health and Care Excellence (NICE) [11]. This itself can tell us the extent of our knowledge in long COVID. More so in paediatrics long COVID, the evidence of the long COVID impact in children is much less compared to adults and appears to be conflicting [13].

Multiple previous studies on long COVID in children, including a systematic review done on symptoms of long COVID in children, did not specifically focus on mental health, as far as our literature search had elicited. The vagueness of the issue is contributed by the fact that the symptoms of long COVID are not specific to the condition, as these symptoms alone or in combination may be present in the general population in absence of long COVID [14]. The elevated mental health symptoms may well be due to the pandemic restrictions and not a direct effect of SARS-CoV-2. This can only be distinguished by studies which have comparison with a normal or non-infected control group of children [15]. A review and meta-analysis on this are critically important as it will delineate the burden of mental health problems specifically experienced by previously COVID-19 infected children. This will help in the allocation of energy and resources appropriate to counter the mental health impacts of the pandemic. Moreover, the symptoms presented in long COVID included symptoms of mental health disorders such as sleep disturbances, depressive symptoms and anxiety which are significant causes of poor quality of life and contribute to the rising global mental health burden [2]. In adults, 30% of patients who had COVID-19 infections were found to have such neuropsychiatric symptoms [16]. Reports on studies of long COVID in children also reported a high percentage of mental health problems, with sleep disturbances up to 33.3% [17] and lack of concentration up to 60.6% [18]. The study by Buonsenso and team which involved 510 children with long COVID, also found high prevalence of mental health related symptoms such as mood changes (58.8%), sleep problems (56.3%) and appetite loss (49.6%) [18]. However, this was an online survey initiated by parents with children affected with long COVID meant to delineate the symptoms prevalent in paediatric long COVID and involved mainly children reporting as having long COVID.

Some studies with control groups have found that the symptoms of long COVID in children were not as high and even were similar to the normal population affected by the pandemic in general [19]. Others have raised the issue that the mental health symptoms such as anxiety are mild changes, arise due to the social and emotional impact following pandemic measures, such as school closures [14]. It is essential to confirm whether mental health problems were increased in children previously infected with COVID-19, or if there was no actual difference compared to non-infected children. This knowledge is crucial to guide decisions and recommendations for policymakers and mental health experts to implement measures to screen and manage these particular conditions if it is significant, therefore ameliorating the

impact on pandemic towards children's mental health. As childhood is a delicate period crucial for the acquisition of psychosocial and behavioural development, having mental health problems at this stage would adversely impact not only their current development, but also their future mental health [20].

Due to the crisis of COVID-19 pandemic and the mental health effects arising from it, WHO has emphasized on the priority and benefits of psychological support and interventions to children and young people. Previous proposed intervention has been suggested to involve skills to regulate emotions and increase activities that promote mental health and wellbeing [4]. However, there is lack of evidence of these activities towards improving depression and anxiety related to the long COVID.

Therefore, this systematic review aimed to assess the proportion of mental health problems post-COVID-19 infection in children and adolescents as the first objective, and to compare these proportion to the proportion of the mental health problems in the normal/non-infected population as the second objective. Conducting proportional meta-analyses are helpful in providing overall estimates for health problems [21], and therefore is applied in this systematic review for the mental health problems in long COVID in children. The paper addresses the question on the proportion of mental health problems, and reports the odds of children infected with COVID-19 having mental health problems as compared to non-infected children; thus, addressing the question of the direct mental health burden from the infection. It also discusses the steps in ameliorating the mental health effects of long COVID, which are in line with the global mental health initiatives to reduce the treatment gap in mental health problems as a way forward as we recover from the pandemic and build back the mental healthcare system better.

Methodology

Comprehensive topic-based strategies designed for each database were used to carry out the search for relevant articles from the following databases: PsycINFO, Scopus, Web of Science (Core Collection), CINAHL, Cochrane Library and EMBASE. The following search strategy is used: (TITLE-ABS-KEY (("Post-COVID-19" OR "post-acute COVID-19" OR "long COVID" OR "ongoing symptomatic COVID-19" OR "post-COVID conditions" OR "post-acute COVID syndrome" OR "long-haul COVID" OR "post-acute sequalae of SARS-COV-2 infection" OR "long coronavirus disease" OR "recovered from COVID-19") AND (children OR adolescents OR pediatric OR paediatric) AND (treatment OR interventions OR management OR prevalence OR survey) AND ("mental health problems" OR "mental problems" OR "anxiety" OR "depression"))). Types of studies included were original articles with observational studies and any intervention trials published from 2019 up to 31st May 2022 with the following inclusion and exclusion criteria:

Inclusion criteria:

a. Observational studies: Cohort studies, case controls, cross-sectional studies, historic cohort studies, or Interventional studies: Randomized controlled trials, non-randomized trials

b. Studies must report the primary outcome: mental health symptoms of long COVID or treatment of anxiety or depression in long COVID among children.

c. Study must be retrievable in the English language.

Exclusion criteria:

a. Reviews, editorials, commentaries, methodological articles, letters to editors, case reports

b. Duplicates/ replicates of studies.

The protocol for reliable and accurate systematic review and meta-analysis of the mental health problems in long COVID among children and treatment approaches for anxiety and depression in long COVID in children was produced and registered in PROSPERO (CRD42022335716).

The population involved in the study was children infected with COVID-19, with long COVID. For pooled prevalence, the comparator group is the normal children population without COVID-19 infection. The definition of children follow the United Nations Convention on the Rights of children, which are those less than 18 years old [22]. The primary outcome is the pooled prevalence, and proportion of mental health problems in long COVID among children with confirmed COVID-19 infection and compared to non-infected population.

In this study, mental health problems are defined as symptoms of mental health problems including but not limited to depression, anxiety, sleep disturbance and cognitive problems [11]. Long COVID is defined as persistent symptoms after 4 weeks of acute infection of COVID-19 (laboratory-confirmed or clinically diagnosed as COVID-19), as defined by the studies.

Study screening and selection

Identified studies were screened independently by two reviewers at five levels:

Level 1: screening of identified studies in the titles and abstracts for entry terms, study design and keywords.

Level 2: further screening of the contents of articles by reading the full article using the same search strategy.

Level 3: snowballing of literature on references from eligible studies.

Level 4: Studies screened at outcome levels to select those that reported the primary outcome.

Level 5: Involved studies reporting primary outcomes.

Conflicts during screening were resolved by a third independent reviewer who served as a tie-breaker.

In the selection process, screened studies were populated into Covidence software, and studies were selected based on study characteristics: study design, inclusion/exclusion criteria, and agreement between two independent and blinded reviewers. Authors of included studies with missing data were contacted via email. Data items were extracted from the selected studies into Excel.

During the data collection phase, data items extracted from selected studies include the surname of first author and year of publication, proportions of mental health problems, instruments used to diagnose or detect the mental health problems, study features such as sample size, study participants and comparison group, study design and assessment time points and treatment or intervention and effect sizes. Data items were exported into a predefined format in Microsoft Excel, and transferred to R and RevMan software for quantitative analysis.

The risk of bias (methodological quality) in the included studies was assessed for each article using the National Institute of Health (NIH) Quality assessment tool for observational cohort and cross-sectional intervention studies. The NIH Quality assessment tool has 14 questions. Studies that score 7 and above are considered good quality. Heterogeneity was assessed

at the study level using the I^2 and T^2. I^2 values of less than 40% were considered low heterogeneity while values > 40 but $< 75\%$ were considered moderate and values $> 75\%$ are high. Significant heterogeneity was also based on the *p*-value for the χ^2 test of heterogeneity. A *p*-value higher than 0.1 indicates insignificant heterogeneity [23].

Random effects model was used in each analysis as this would allow meta-analysis to be done in the presence of high heterogeneity by assumption of the normal distribution [24]. Funnel plots were used in some cases to examine for bias. A symmetrical inverted funnel means that there was absence of bias and heterogeneity is due to sampling variation [25].

Strategy for data synthesis

Extracted data items were used for both narrative synthesis and quantitative analysis.

The following criteria were applied for analysis:

a. Studies that passed the methodological quality assessment using the NIH quality assessment tool were analysed and presented in tabular format, indicating all the extractable data items as listed under data collection.

b. All studies with primary outcome were included for quality analysis.

c. All studies with good NIH quality scores that reported the outcomes were used for quantitative synthesis.

Quantitative analysis, which was meta-analysis for pooled prevalence and comparison between children with previous COVID-19 infection with non-infected children was performed with the available studies. However, due to the limited studies having the data required with high quality and homogeneity, all studies with moderate to high quality assessment reports were included for the pooled prevalence analysis and comparison. Minimum two studies are needed, as meta-analysis means two or more studies results on the same issues are analysed using a set of technique [26]. Pooled prevalence for each of the mental health outcome was estimated by meta-analysis using a Freeman-Tukey double arcsine transformation and a random effects model using the *metaphor* package in R software. The computational analysis used was the random effects model as studies included in the meta-analyses were from different children populations all over world and had moderate to high heterogeneity in the majority of the analyses. Meta-regression or subgroup analysis was not performed on quantitative variables due to the limited studies included in the meta-analysis for each mental health problem. Incidence comparisons for each mental health problems were done between children with previous COVID-19 infection and non-infected children, with odds-ratio reported as it can be used in both cohort and case-control studies [27]. A two-sided *p*-value <0.05 was considered significant. Cases were children with COVID-19 infection, while control were children with no previous COVID-19 infection.

Analyses were done for five mental health problems or symptoms as outcomes elicited from the studies, namely:

i) Anxiety

ii) Depression

iii) Difficulties in concentration

iv) Sleep problems

v) Mood swings

vi) Appetite problems

Results

The total number of studies identified from January 2019 to May 2022 was 1848. Automated removal of duplicates was done by Covidence, removing 193 records. Another three duplicates were manually excluded, and another 1581 records which did not fulfil the inclusion and exclusion criteria during the screening of topics and abstracts were also excluded. Subsequently, 71 full texts were screened, including five papers from hand searches of references. A total of 27 papers were excluded as they focused on adult populations, which was not mentioned in the abstract, 18 were excluded as they did not have outcome data such as non-mental health outcome, 10 had other study design such as being reviews, two were excluded due to the full text being in other languages, both Russian, and one was excluded as it was a preliminary report on a study which was already included. Finally, 13 studies were included in the quality rating analysis. Fig 1 shows the flow of records selection according to PRISMA flow chart [28, 29].

Except one study from China, no other included studies came from developing countries. Two studies were from the United Kingdom, one from Sweden, two from Denmark, one from Germany, one from Switzerland, one from Russia, one from Latvia, two from Italy, and one from Wuhan, China.

The majority of the studies used RT-PCR (Reverse Transcriptase-Polymerase Chain reaction) as a confirmatory test to diagnose COVID-19, except for three studies. RT-PCR is the gold standard test for diagnosing COVID-19, which detects the virus itself. Two studies by Blankenburgh and team [30], and Radtke and team [31] used serological tests (test for antibody for SARS-CoV2) for all the participants, while another study included some proportion of samples tested using serological test [19]. In these studies, the results, in general, did not show any difference in the proportion of mental health symptoms between serological positive and negative groups. As the diagnosis for COVID-19 using serological test is not accurate, where it was found that almost 20% of adults who had a positive RT-PCR for COVID-19 were not seropositive [32], and due to other biases existing in the studies, the two studies which used totally serological tests were excluded from quantitative analysis.

Most studies also used parents or guardians as proxies to their children. Only two studies had the adolescents themselves answer the questionnaire: a study from Denmark [13] and a study from England [33]. One study from Latvia did a face-to-face interview with the patients and carers [34]. In addition, the majority of the studies were community studies, while three studies observed hospital-discharged children, one from China [35], one from Russia [36] and one from Sweden [37]. In general, these studies did not necessarily differ significantly in mental health problem proportions as compared to community studies. For example, the study by Sterky and team [37] with a sample discharged from two Stockholm paediatric hospitals had a depression rate of 5.5% as opposed to a community study by Zavala and team, with 3.8% depression rate in the studied population [38].

(S1 Table) summarizes the number of studies pooled for each symptom and the assessment tools, sample sizes, assessment times and populations. It can be seen from the table that the studies indeed have distinct variations from each other, thus contributing to the high heterogeneity in the analyses.

Following assessment for risk of bias and quality, five studies were rated as good quality or low risk of bias, while six studies were rated as moderate quality or moderate risk of bias. However, due to the limited studies available having moderate to good quality, each study would be valuable to give an estimate for each of the mental health symptom or problem, thus included in the quantitative analysis. Two of the studies were not included in the quantitative analysis

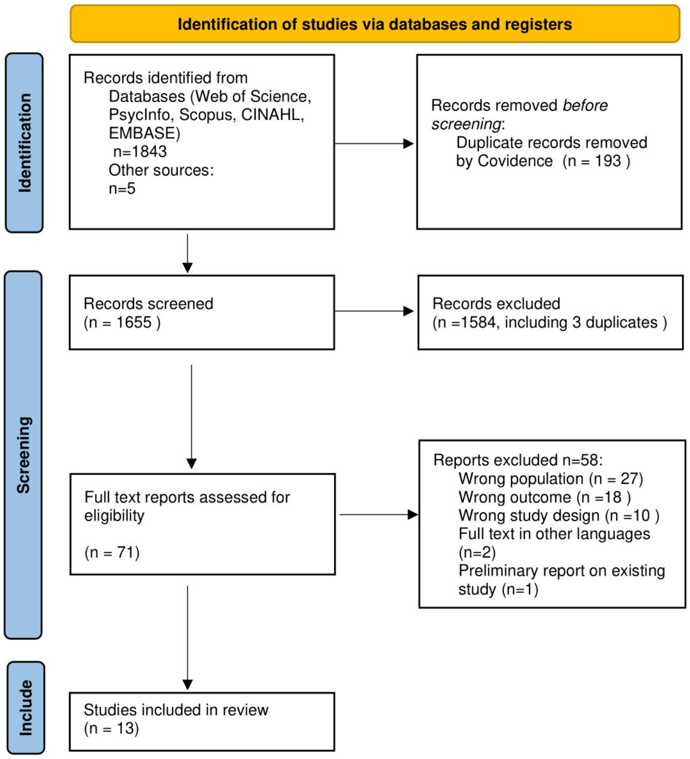

Fig 1. PRISMA flowchart for studies selected for review.

https://doi.org/10.1371/journal.pone.0282538.g001

due to being rated low quality or high risk of bias (Table 1). Example of the risk of bias and quality assessment for the studies is provided in S2 Table.

Results were reported for the two types of analyses which were done for each mental health problem. First type was to determine the proportion of mental health problems in long COVID, where a proportional meta-analysis of the outcome was calculated from all studies which provided available data for the outcome, and the second type was a meta-analysis of odds-ratio for outcome between cases and controls in controlled studies. A summary of pooled proportion results is featured in S1 Table.

i) Anxiety

Three studies had information on anxiety and were included. Pooled proportion for anxiety from studies which had anxiety as an outcome showed a 9% prevalence of anxiety in long COVID among children who had a COVID-19 infection (Fig 2). The heterogeneity was high and the random effects model was used.

Although the percentage appear to be small, the comparison of anxiety in long COVID proportion among children with COVID-19 infection to non-infected children controls showed that it is significantly different. Children with previous COVID-19 infection were at 2.11 higher odds of having anxiety in long COVID as compared to children without COVID-19 infection (Fig 3). Funnel plot done with symmetry suggests that any heterogeneity of the studies were related to sampling differences (Fig 4).

Table 1. Description of studies included and outcome of risk of bias/ quality assessment of the studies.

No	Study (First Author, year, site)	Sample size	Study participants	Study design	Sampling and measuring tools	Comparison	Assessment time point	Findings	Outcome of risk of bias/ Quality assessment
1	Blankenburg, 2022, Germany	1560 (188 cases and 1365 controls)	Grade 8–12, median 15 years old	Cohort, proxy-reporting by parents	Samples taken from students enrolled in a regular serological status assessment for a survey called SchoolCOVID19 study. A 12 question Long-COVID19 survey conducted to assess neurocognitive, general pain and mood symptoms.	Seropositive adolescents versus seronegative at different timepoints.	Survey was done assessing symptoms 7 days prior in March 2021, with seroprevalence assessment done in 3 timepoints, in May 2020, October 2020, and March 2021.	More than one third ofadolescents experience one symptom assessed in Long-COVID19 survey. Symptoms presences were not significantly different in both groups except for the seropositive being less sad. Comparing seropositive and seronegative:<br>• Difficulty concentrating: 80.9% vs 79.1%<br>• Sleep problems: 62.9% vs 66.9%<br>• Sad mood: 57.9% vs 66.3%	Excluded for quantitative analysis due to low quality rating. Criteria for assessing positivity using antibody test is debatable. Seronegative adolescents may have a previous infection. For seropositive, the time point of infection could not be assessed and may be less than one month infected.
2	Berg, 2022, Denmark	28270 (6630 cases, 21640 controls)	Adolescents aged 15–18 years 2021 Median age: 17·6 years (IQR 16·4–18·5) Females: 57·6% of 28 270	Cohort study, adolescents to answer the online questionnaire.	All adolescents with a positive SARS-CoV-2 test from 1st Jan 2020 to 12th July 2021 identified from a national COVID-19 database and a group of controls were sent a survey in their digital post-box from July 20, 2021 to Sept 15, 2021. Symptoms associated with COVID-19, school attendance, comorbidities and health-related quality of life were investigated using ancillary questions and validated questionnaires (Paediatric Quality of Life Inventory [PedsQL] and Children's Somatic Symptoms Inventory-24 [CSSI-24]) and 23 common long COVID symptoms. Long COVID identified from symptoms lasting two months.	Control group matched (1:4) by age and sex identified from national registry, those who had a known or suspected COVID-19 infection or infected during the data collection were excluded.	Time of assessment from testing varied from less than one month to more than 12 months. Persistent symptoms defined as symptoms lasting at least 2 months.	2997 out of 6264 had long COVID (47.8%).<br>Most frequent symptoms: headache, fatigue, loss of appetite, trouble breathing and trouble concentrating or remembering.<br>When comparing positive group to the control group:<br>• Mood swings: 554 (10.9%) vs 2756 (12.7%).<br>• Trouble concentrating or remembering: 616 (12.1%) vs 2636 (12.2%).<br>• Loss of appetite 403 (7.9%) vs 1498 (6.9%).<br>Positives had higher odds of long-lasting symptoms (OR 1.22, 9%% CI 1.15–1.30, p <0.001), more sick days and school absences but had better quality of life scores and lower somatic distress compared to controls.	Included with good quality rating. However,there was some risk of bias due to the assessment timepoints from SARS-CoV-2 positivity varied in samples, up to 12 months retrospective recall, may have caused recall bias from those with longer duration.

(*Continued*)

Table 1. (Continued)

No	Study (First Author, year, site)	Sample size	Study participants	Study design	Sampling and measuring tools	Comparison	Assessment time point	Findings	Outcome of risk of bias/ Quality assessment
3.	Borch, 2022, Denmark	31021 (15941 cases and 15080 controls)	Danish children age 0–17 years<br>Cases:<br>- 39.1% 6–17<br>-mean age 12<br>Control:<br>-18.3% 6–17<br>-mean age 10.5	Cohort study, proxy-reporting by parents, online questionnaire	National cohort with cases samples taken from Danish Health Data Authority list of all Danish children aged 0–17 with RT-PCR verified COVID-19 infection between Jan 27th 2020 to March 19th 2021. Questionnaire ask for symptoms lasting more than 4 weeks, demographic and chronic disease data, WHO-5-well-being index	SARS-CoV-2 positive (RT-PCR) versus control group who are recruited via schools and daycare in five municipities in Denmark without previous known infection.	Electronic questionnaire was sent from March to May 2021 with 4 weeks response time. Those with PCR tests less than 4 weeks were excluded.	For symptoms more than 4 weeks: 28% of SARS-CoV-2 positive, 27.2% in controls, despite having higher sense of wellbeing evidence by higher WHO-5 score (therefore symptoms not due to the pandemic psychological consequences). Older children more.<br>• cognitive difficulties present in 6.1% cases versus 9.2% in controls.	Included with good quality rating. However, the assessment timepoints from SARS-CoV-2 positivity varied in samples, may have caused recall bias from those who had earlier tests.
4.	Molteni, 2021, United Kingdom	3468<br>(1734 out of 6975 who had a positive COVID-19 test and 1734 random matched negative children).	UK children 5 to 17 years old, with an adult proxy logging in a mobile app.<br>Cases:<br>Median 13 (IQR 10–15)<br>Males: 49.8%<br>Controls<br>Median: 13<br>(IQR 10–15)<br>Males: 49.9%	Cohort study. Data reported by parents in a mobile app.	Data obtained from adult proxy voluntary participants, reporting for children in a mobile app, where daily symptoms, COVID-19 tests results and healthcare access were recorded. Data from app for children with illness onset from Sept 2020 to 22nd Feb 2022 were analysed.	Children who had proxy-adults reporting symptoms on the app testing negative for SARS-CoV-2, matched 1:1 for age, gender, and week of testing.	Real-time collected data through a digital surveillance platform analysed. (during first week, first 28 days, from day 28 until illness end, and entire illness duration).	Symptomatic COVID-19 infection reported in the app for children aged 5 to 17 is short (6 days) and prolonged duration 28 days or more was 4.4%. Cases had prolonged illness duration than controls (0.9%), p<0.001.	Include with moderate quality rating.<br>The limitations detected were:<br>• lack of representativeness (selection bias due to nature of data collection)<br>• Response bias (only 25% cases regularly logged data)<br>• assessed children with defined illness duration and recovery was defined as reporting asymptomatic or cessation of logging, which would exclude children with symptoms not having logged in or had a break in the symptoms.
5.	Osmanov, 2021, Russia	518 of 853 patients.	Children less than 18 years old. Median age 10.4, 52.1% girls	Prospective study. Interview parents. Follow-up five months after discharge	Children discharged from Bashlyaeva Children's Municipal Clinical Hospital, admitted from 2nd April 2020 to August 2020 with RT-PCR confirmed COVID-19 infection were followed-up up to 7 months post-discharge.	No control group.	Parents contacted between 31st Jan 2021 to 27th February 2021, seven to nine months post discharge.	24.3% had persistent symptoms of more than 5 months: fatigue (10.7%), sleep disturbances (6.9%), sensory problems (5.6%), confusion or lack of concentration (0.41%)	Included in prevalence study with good quality rating. However, recall bias was likely due to prolonged time between testing and assessment.

(*Continued*)

Table 1. (Continued)

No	Study (First Author, year, site)	Sample size	Study participants	Study design	Sampling and measuring tools	Comparison	Assessment time point	Findings	Outcome of risk of bias/ Quality assessment
6.	Clavenna, 2022, Italy	148, 41 cases 107 controls	Children aged 0 to 16 years old. Median age: 6.5 (IQR:3.5–10.5)	Prospective study, follow-up 6 months.	Children with suspected COVID-19 infection from February 2020 to June 2020 and underwent testing (either molecular or/ and antibody) were enrolled by outpatient paediatricians in Lombardy region, collected information on demographics, symptoms, diagnostic tests and presence of comorbidities. Follow-up of symptoms after 6 months post first consultation. Checklist for symptoms with yes/no answers.	Children who tested positive (n = 41, 15 molecular testing versus children tested negative for COVID-19 (n = 107, 86 molecular testing, 21 serological.	During acute illness and 6 months after.	24 children (17%) had psychological distress, which was not present before the epidemic; (cases versus controls)<br>• sleep disorders: 4 (9.8%) versus 12 (11.2%)<br>• anxiety 1 (2.4%) versus 5 (4.7%)<br>• irritability 1 (2.4%) versus 6 (5.6%).	Moderate quality rating. Included in the pooled analysis with caution due to the categorization of a proportion of the children were done by using serological test.
7.	Buonsenso, 2021, Rome, Italy	129 children with COVID-19.	All children less than 18 years old diagnosed with COVID-19 using RT-PCR, without severe neurological disability, mean age 11 years old, 48.1% female	Cross-sectional, caregiver interviewed	All patients tested positive in Fondazion Policlinico Universitario A. Gemelli IRCC from March to October 2020 (at least 30 days of diagnosis) were included. Participants were interviewed face to face in the outpatient department or via call from 1 sept 2020 to 1 Jan 2021, using questionnaire developed by Long COVID ISARIC study.	No control group.	Minimum 30 days from diagnosis.	58.1% had at least one persisting symptom. Insomnia: 24 (18.6%)	Included for pooled prevalence with moderate quality rating.
8.	Roge, 2021, Latvia	378 (236 cases and 142 controls)	1 month to 18 years old median age 6 IQR 2–12 Cases:10.0 (5–14) Controls: 2.0 (1–6)	Cohort study Patients or their parents/ caregivers/legal guardians were interviewed "face-to face".	Invitation via media and medical facilities to families with children recovered from COVID-19 infection. Controls were recruited from the patients who were treated at the hospital and clinics in Latvia. Used a specially designed post-COVID-19 symptom assessment questionnaire, enrolled between 1st July 2020 to 30th April 2021 for cases, and between 1st November 2020 and 30th April 2021 for controls.	COVID-19 infection versus Non-SARS-CoV-2 community-acquired infections with no history of COVID-19 and clinical and laboratory findings confirming other infections.	Eligible subjects or their parents/ caregivers/legal guardians were interviewed 1–6 months post infection.	70% of cases reported at least one persistent symptoms:<br>• Difficulties to concentrate: 16.9%<br>• Depression/ Anxiety: 13.1%<br>• Mood changes: 23.3%<br>• irritability: 23.3%. Children post-COVID-19 who were hospitalized were significantly more frequent in having catarrhal symptoms	Included for quantitative analysis with moderate quality rating. Cases prone for selection bias as volunteers may be from those still having symptoms. It was noted also that the comparison group was younger, median 2 years old versus median 10 for cases. Anxiety and depression were lumped together and not assessed according to criteria.

(*Continued*)

Table 1. (Continued)

No	Study (First Author, year, site)	Sample size	Study participants	Study design	Sampling and measuring tools	Comparison	Assessment time point	Findings	Outcome of risk of bias/ Quality assessment
9.	Stephenson, 2022, United Kingdom	6804	11 to 17 Adolescents tested PCR for COVID-19 between Jan to March 2021 contacted 3 months post test.	Cohort, Children answer themselves with help from carer	Adolescents answer an online questionnaire about their physical and mental health using ISARIC paediatric COVID-19 and Mental Health of Children and Young people in England questionnaire, Warwick Edinburgh Mental Wellbeing Scale (SWEMWBS) used to assess wellbeing, EQ-5D-Y for quality of life, CFQ for fatigue, TD score from SDQ used for behavioural problems before COVID-19 infection and at the time of completing the questionnaire.	Adolescents aged 11–17 years with PCR-confirmed SARS-CoV-2 infection compared with matched adolescents with negative PCR status.	3 months after testing.	At three months post test, 2038 (66.5%) of cases and 1993 (45.3%) controls had any kind of symptoms. For both groups, symptoms were higher in the 15–17 years group compared to the younger group. Symptoms related to mental health (cases versus controls): Skipping meals: 360 (11·7%) versus 67 (1·8%) Confusion or difficulty in concentration: 198 (6.5%) versus 123 (3.3%).	Included for quantitative analysis with good quality rating. Mental health wellbeing assessed using Likert scale. Unable to compare with other studies.
10.	Zhang, 2021, Wuhan, China	152 (61 cases 91 controls)	7 to 18. Cases from hospitalized children with COVID-19 from January to March 2020. Cases: 27.8% 13–18 years old 78.7% males Controls: 7.7% 13–18 years old 55% males	Case control Hospital	Children hospitalized for COVID-19 in Wuhan Children's Hospital (diagnosed based on criteria of national guideline) were recruited through call, assessed from July to September 2020. Child PTSD Symptom Scale (CPSS), Screen for Child Anxiety Related Emotional Disorders (SCARED), 10-item Children's Depression Inventory-Short version (CDI-S), 26-item Sleep Disturbance Scale for Children (SDSC), 10-item Post-traumatic Growth Inventory–Short Form (PTGI-SF), Brief Trauma Questionnaire (BTQ), Connor–Davidson Resilience Scale (CD-RISC-10), 12-item Multidimensional Scale of Perceived Social Support (MSPSS)	Control group recruited from a birth cohort in Wuhan Children's Hospital, assessment done during the same period.	Four months after discharge	The prevalence of PTSD, anxiety, and depression was found to be higher in discharged children who recovered from COVID-19 compared with healthy controls (PTSD: 8.20 vs. 2.20%, p = 0.084; anxiety: 22.95% vs. 13.19%, p = 0.12; depression: 47.54 vs. 32.97%, p = 0.082) Also determined the factors associated to mental health problems in discharged patients: presence of PTSD and poor sleep in caregivers.	Included for quantitative analysis with assessment of moderate quality. Sampling bias exist during selection of cases as it is non-random. Out of 363 patients hospitalized, 243 were excluded due to not being contacted, 55 due to less than 7 years old and 4 incomplete, leaving 61 cases. Control group were not matched with age, had less adolescents compared to cases.
11.	Sterky, 2021. Sweden	55	Children aged 0–18 years old admitted to two Stockholm paediatric hospitals due to COVID-19 with RT-PCR positive. 58% males.	Prospective	Children hospitalized from 13 March to 31 August 2021 due to COVID-19 were enrolled, 55 of 60 were finally included, two lost to followup 3 not consented. Depression was asked as a yes/ no single symptom.	No control group	At admission and followed up at four to 10 months post discharge	12/55 (22%) had persistent symptoms at four months. 3 (5.5%) had cognitive difficulties. 3 (5.5%) had depression.	Moderate quality rating. Included for quantitative analysis of pooled prevalence with caution due to assessment being vague. Follow-up assessment time too variable from four months up to ten months post discharge.

(*Continued*)

Table 1. (Continued)

No	Study (First Author, year, site)	Sample size	Study participants	Study design	Sampling and measuring tools	Comparison	Assessment time point	Findings	Outcome of risk of bias/ Quality assessment
12.	Zavala 2021 England, United Kingdom.	859 (472 cases and 487 controls)	Children 2 to 16 years old with a positive or negative PCR test from 01–07 January 2021	Cohort, proxy reporting by parents/ guardian	National testing data utilized to identify children aged 2–16 years with a SARS-CoV-2 PCR test from 01–07 January 2021, randomly selected 1,500 PCR-positive cases and 1,500 matched PCR-negative controls using stratified random sampling (by age and postcode). Letters sent out on 22nd February 2021 inviting parents to fill online questionnaire for demographics, COVID-19 symptoms at the time of testing, household composition, confirmed cases in the household and pre-specified symptoms at the time of the RT-PCR test and at least one month later.	Children with a negative PCR test during 01–07 January 2021	Minimum one month post testing. Ongoing symptoms defined as minimum five times experience at least one month post-test.	Higher prevalence of acute (68% vs 40%) and on-going symptoms at 1 month (6.7% vs 4.2%) in children with PCR-confirmed COVID-19 compared to PCR negative symptomatic controls, but mental health symptoms were high and prevalent in both groups.<br>For PCR positive vs PCR negative:<br>• anxiety 7% vs 2.3%<br>• sadness 5.7% vs 1.0%<br>• mood swings 6.6% vs 2%<br>• depression 3.8% vs 1%;<br>• difficulty sleeping 7% vs 2.3%<br>• confusion 4% vs 0.4%	Included for quantitative analysis with good quality rating.<br>However, depression and anxiety symptoms were asked as a yes/ no single symptom.
13.	Radtke, 2021, Switzerland	109 seropositive and 1246 seronegative (1355 of 2503 eligible children)	6 to 16 years old	Cohort, proxy reporting by parents	Students from 55 randomly selected schools from randomly selected classes to participate, serological tests were done in October and November 2020 –those seropositive were compared with those seropositive. March 2021 to May 2021 parents filled Questionnaire of symptoms occurred since October 2020 and lasting for 4 weeks and 12 weeks. Questionnaire with list of predefined symptoms and free text.	1246 seronegative school children	4 months after last serological test.	Comparing seropositive versus seronegative children; 9% versus 10% report persisting symptoms at 4 weeks, and 4/109 (4%) versus 28/ 1246 (2%) reported at least 1 symptom persisting symptoms at 12 weeks.	Not included for quantitative analysis due to low quality rating<br>Prone for recall bias and diagnostic misclassification. Seronegative group had higher percentage of older age children.

https://doi.org/10.1371/journal.pone.0282538.t001

ii) Depression

As for depression, three studies had depression as an outcome. The pooled prevalence showed a figure of 15% (95% CI: 0.4, 46) (Fig 5). The analysis showed high heterogeneity among studies and random effects model was used. Only two of the studies had a control group and was included in further analysis. Children with a previous COVID-19 infection also had more than two times higher odds to have depression compared to those without (Fig 6).

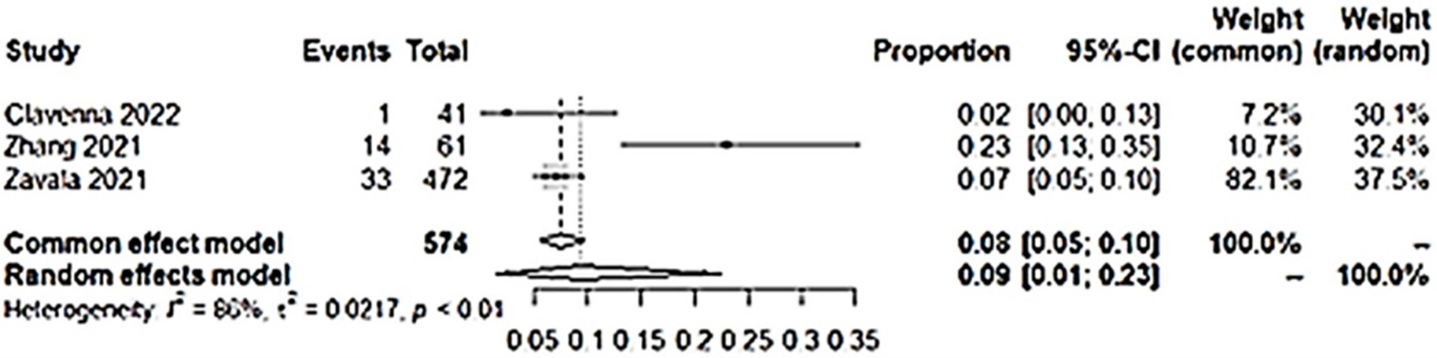

Fig 2. Pooled prevalence of anxiety among included studies.

https://doi.org/10.1371/journal.pone.0282538.g002

iii) Difficulties in concentration

Seven studies reported concentration difficulties as an outcome in long COVID among children. Pooled prevalence showed a figure of 6% (Fig 7). There was no significant difference in the incidence of concentration problems among children with a previous infection with COVID-19 than those without (Fig 8).

iv) Sleep problems

Five studies included sleep problems as an outcome in long COVID among children. Pooled prevalence showed a figure of 9% (Fig 9). Three studies had a control group and were included in the subsequent analysis. There was no significant difference of incidence of sleep problems among children with a previous infection with COVID-19 than those without (Fig 10).

v) Mood swings

Three studies had mood swings as an outcome, and all had control groups. Pooled prevalence showed a figure of 13% (Fig 11). There was no significant difference of incidence of mood swings among children with a previous infection with COVID-19 than those without (Fig 12).

iv) Appetite problems

Three studies had mood swings as an outcome, and all had control groups. Pooled prevalence of studies which had appetite problems as an outcome showed an output of 5% (Fig 13).

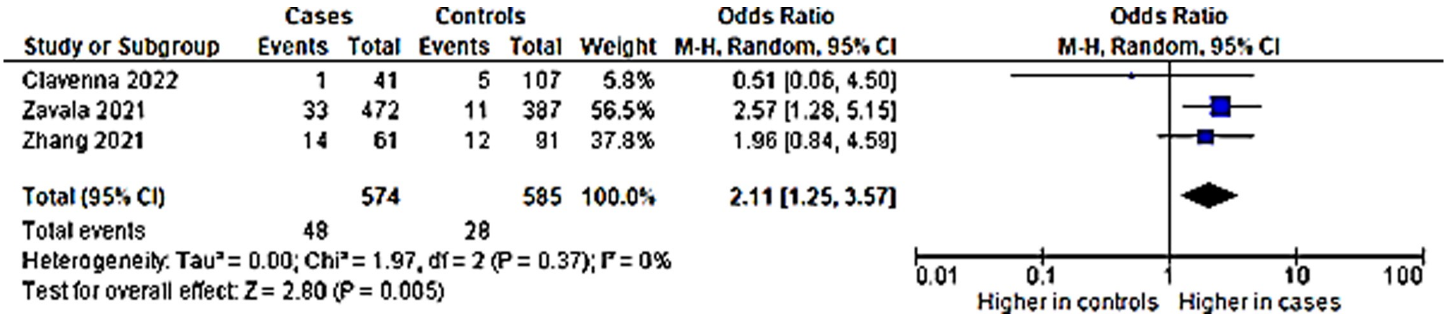

Fig 3. Anxiety in long COVID among children with previous COVID-19 infection versus controls.

https://doi.org/10.1371/journal.pone.0282538.g003

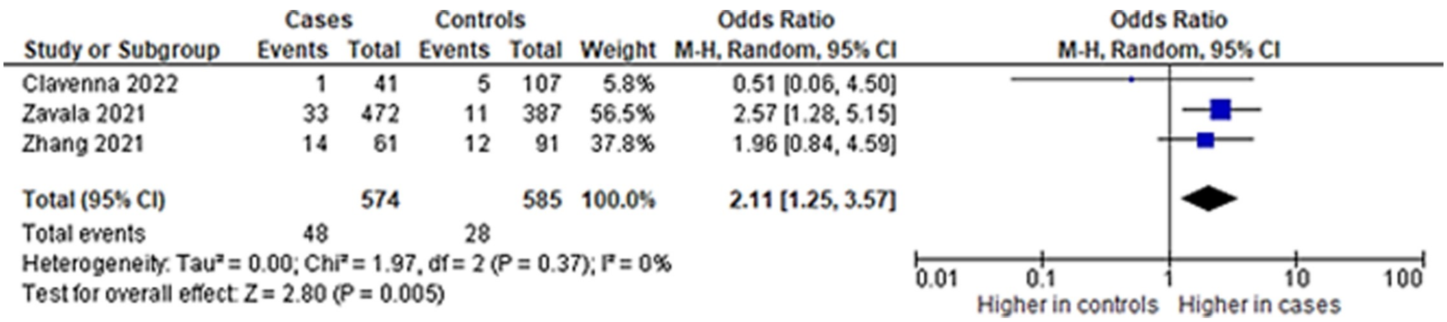

Fig 4. Funnel plot suggesting the heterogeneity of the studies are related to sampling differences.

https://doi.org/10.1371/journal.pone.0282538.g004

Although the percentage seems low, the children with previous COVID-19 infection are at 14% higher odds of having appetite loss in long COVID as compared to children without COVID-19 infection (Fig 14). Funnel plot with acceptable symmetry suggesting the heterogeneity among studies were related to sampling differences (Fig 15).

Discussion

This systematic review and meta-analysis provide timely estimates of the prevalence of important mental health problems or symptoms in long COVID among children with COVID-19 infection, and evidence regarding the effect of increased mental health problems occurrences in long COVID. The evidence that children with previous COVID-19 infection are more than two times at higher odds of having anxiety and depression underscores the importance of timely assessments of children with long COVID for mental health problems and early interventions.

Due to the lack of a clear definition of long COVID, there were differences in the timing of the studies conducting the assessments of long COVID. WHO has defined long COVID as post-COVID-19 condition, with symptoms at three months from the onset of COVID-19 that last at a minimum of two months, unexplainable by other alternatives [12]. However, NICE has decided to consider symptoms after four weeks of COVID-19 diagnosis sufficient to

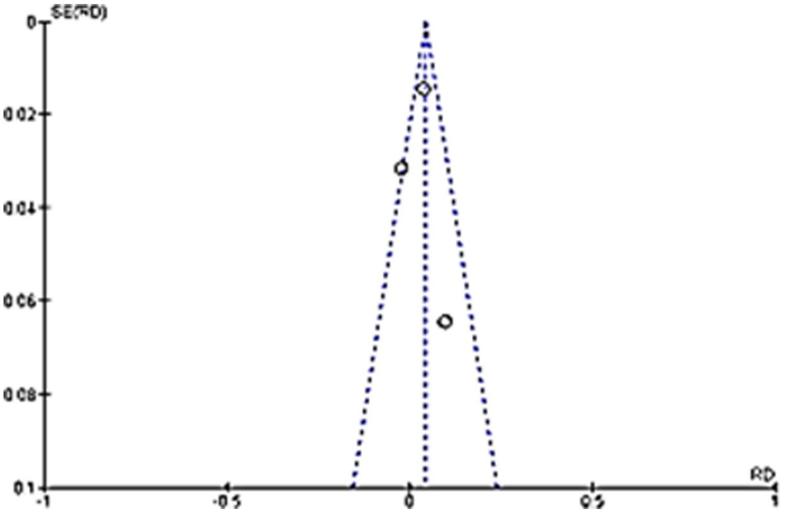

Fig 5. Pooled prevalence of depression among included studies.

https://doi.org/10.1371/journal.pone.0282538.g005

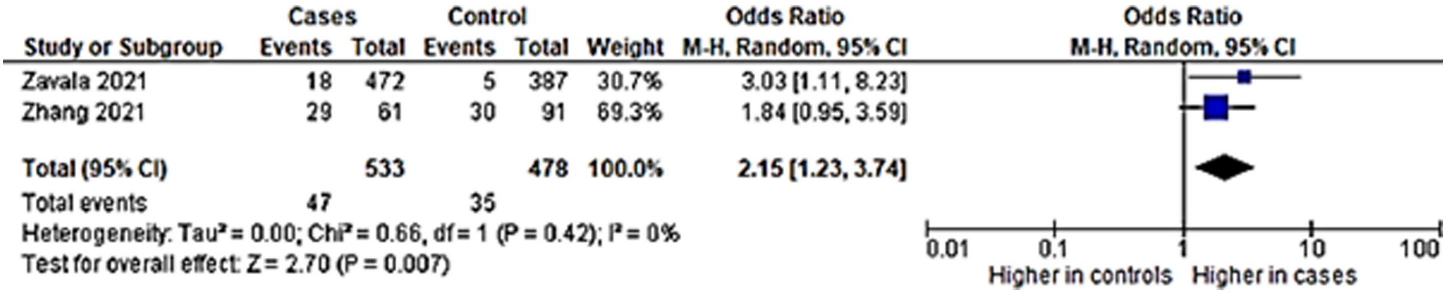

Fig 6. Depression in long COVID among children with previous COVID-19 infection versus controls.

https://doi.org/10.1371/journal.pone.0282538.g006

suspect long COVID [11]. The diagnosis of COVID-19 itself in studies needed to be accurate in order to establish a diagnosis of long COVID. Serological tests for COVID-19 which detect the antibody produced by the immune system following infection with the SARS-Co-V2 virus has been deemed by the United States Food and Drug Administration (FDA) as incapable of diagnosing a current or previous COVID-19 infection for a few reasons. Firstly, a false negative test might occur, which means that the test did not detect the antibody even though antibodies were present [39]. Secondly, a person's body might not produce antibodies detectable by the test, even though the person was infected. In adults, it was shown that 18% of those infected do not produce antibodies [40]. Even when the body did produce antibodies, the level might not be high enough or may wane by the time the assessment was done. The accuracy of the serological tests to diagnose COVID-19 also varies widely, and can only be sensitive enough to detect up to 90% of people after being infected for 15 days [41]. Despite this, two cohort studies have used serological testing to diagnose long COVID (Table 1). Therefore, the results of these studies would be confusing to be interpreted as both of these studies showed that serological positive and negative groups had similar rates of symptoms, which had been interpreted to be the evidence of absence of long-term COVID-19 impact [14].

Previous analyses have noted that the timing of assessment in long COVID to be significant in influencing the presence or absence of symptoms as the mental health symptoms appear to have a higher prevalence in the longer assessment studies. Therefore, the timing of assessments in long COVID is very important in order to determine an accurate estimate of incidence of

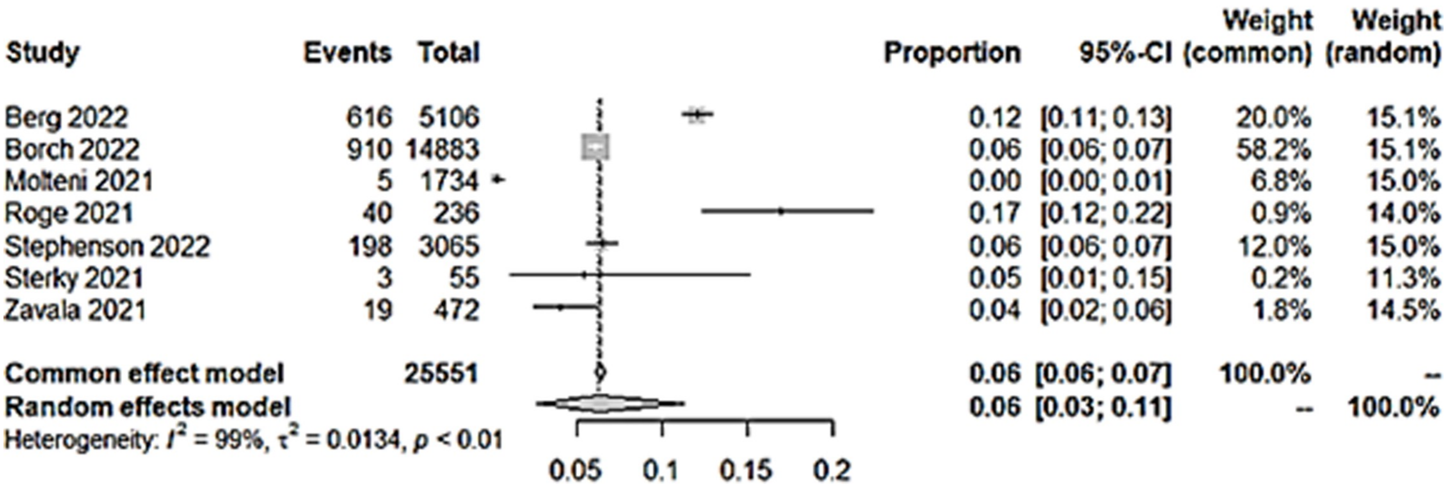

Fig 7. Pooled prevalence of concentration difficulties in long COVID among children infected with COVID-19.

https://doi.org/10.1371/journal.pone.0282538.g007

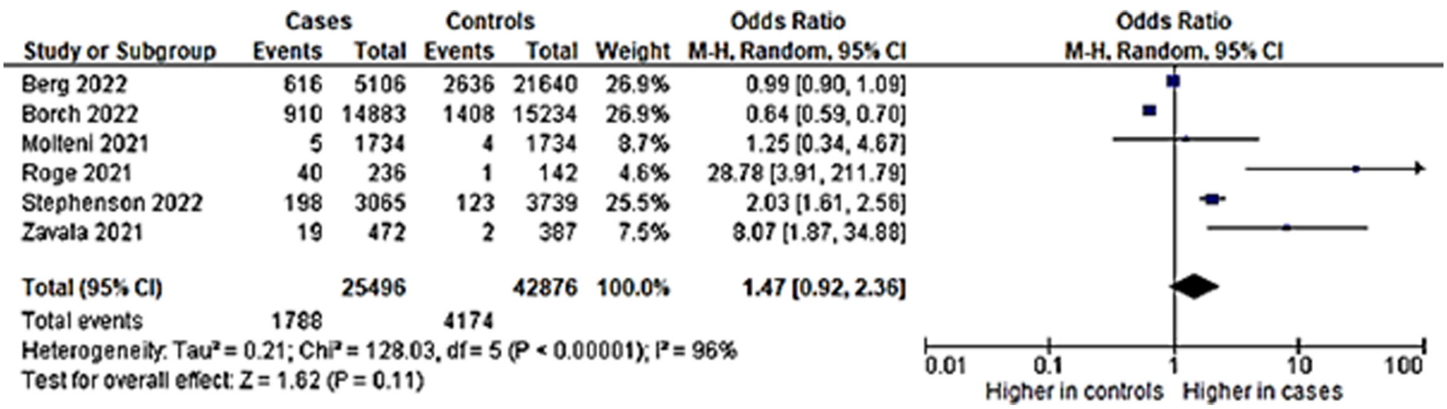

Fig 8. Difficulties in concentration in long COVID among children with previous COVID-19 infection versus controls.

https://doi.org/10.1371/journal.pone.0282538.g008

mental health problems in long COVID. As seen in Table 1, a variety of time intervals were used during the assessment of long COVID symptoms which may have contributed in a varying degree to the different incidence of mental health problems. It was found that mental health problems such as depression and cognitive defects were more pronounced after one to four months post diagnosis [42].

Previously, it was unclear of how much mental health problems have intensified due to the SARScoV2 infection itself, rather than other effects of the pandemic. The estimates of pooled prevalence for mental health problems among children with a previous COVID-19 infection ranged from 5% (95% CI: 1,13) for appetite problems to 15% (95% CI: 0.4, 47) for depression. The estimates were higher than previous estimated global pooled prevalence of mental disorders among children less than 18 which was 13.4% for mental health problems, 6.5% for anxiety disorders and 2.6% for any depressive disorders [6]. However, it was lower than the pandemic estimates which were 20.5% and 25.2% for anxiety and depression [5]. The contribution of mental health problems from long COVID appear to be higher for depression compared to anxiety.

The studies found in this systematic review specifically assessing anxiety and depression in long COVID are limited and the methodologies applied were various, causing significant heterogeneity for the pooled prevalence estimate. For anxiety in long COVID, only three studies

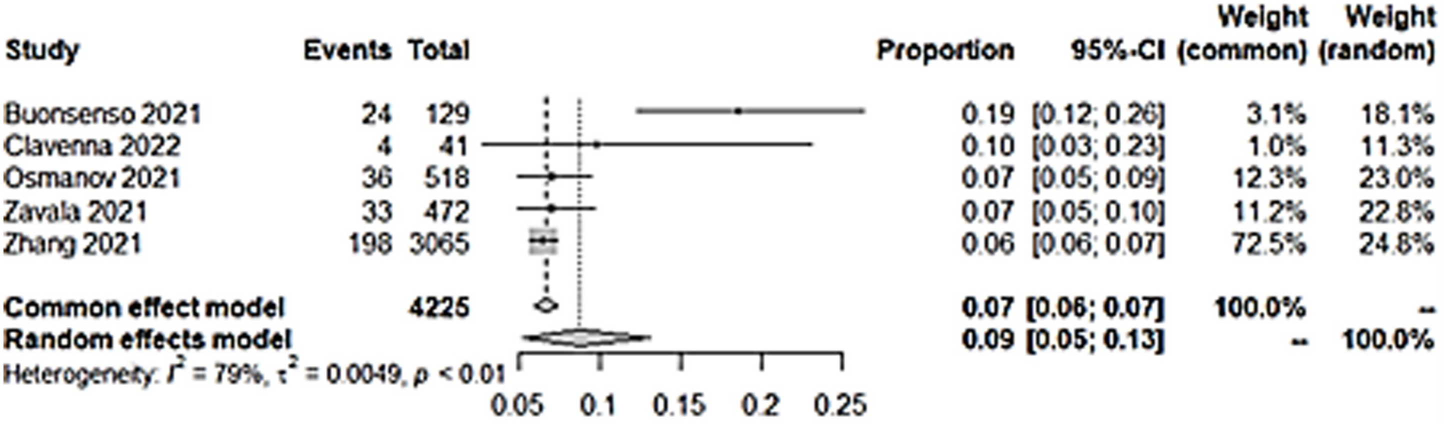

Fig 9. Prevalence of sleep problems in long COVID among children infected with COVID-19.

https://doi.org/10.1371/journal.pone.0282538.g009

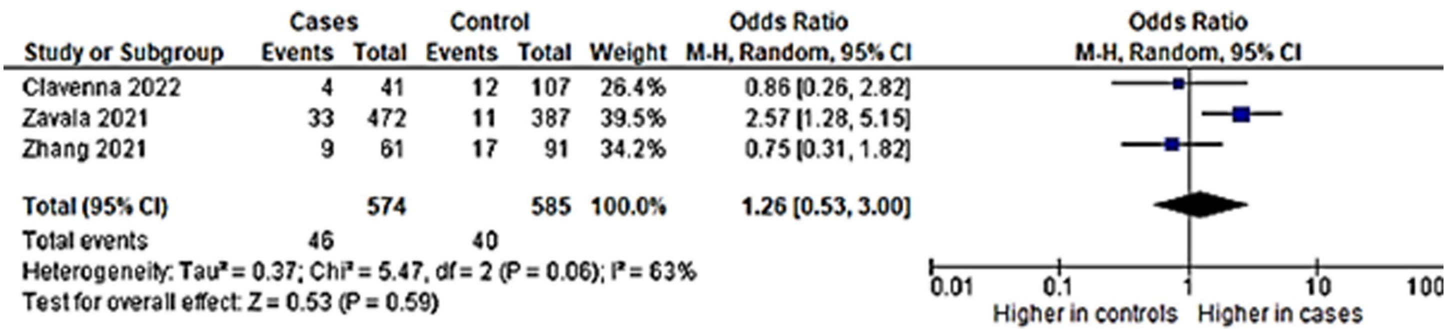

Fig 10. Sleep disturbances in long COVID among children with previous COVID-19 infection versus controls.

https://doi.org/10.1371/journal.pone.0282538.g010

had assessed anxiety together with a comparison group. The study by Zhang and team was the only study that mentioned using a validated questionnaire dedicated to assess anxiety in children and yielded a proportion of 23% [35]. However, the population of cases was children hospitalized in Wuhan Children's Hospital, with the assessment time between four to six months. The lowest proportion of children with anxiety was from a study in Italy, which has a follow-up of six months post COVID-19 infection. This may show that hospitalized children had more anxiety, and this condition was more pronounced at the minimum period of four months post-infection.

The influence of assessment period was supported by studies in adults which acknowledged that mental health problems were more apparent between one to four months [42]. However, the assessment tool also might play a part in detecting more anxiety cases. In the study by Zhang et al., the proportion of the normal population control group having anxiety was 13.2% [35], which was higher than among the cases for the other two studies, which were 7% [38] and 2% [19] (Fig 1). Therefore, it is evident that usage of the screening test is more sensitive to detect anxiety. The timing of study during different phases of the pandemic could be influential as well, as Zhang conducted the study earlier in the pandemic, while the national study in England by Zavala was done among children who were positive in January to February 2021. The study by Zavala was done during the wave of the alpha variant in England, which may affect the risk and prevalence of long COVID [38].

As for depression, the study with the highest rate of depression was from Wuhan, where almost half of the children who were discharged from Wuhan Children's Hospital had depression at four months. This study used a validated questionnaire specifically to assess depression,

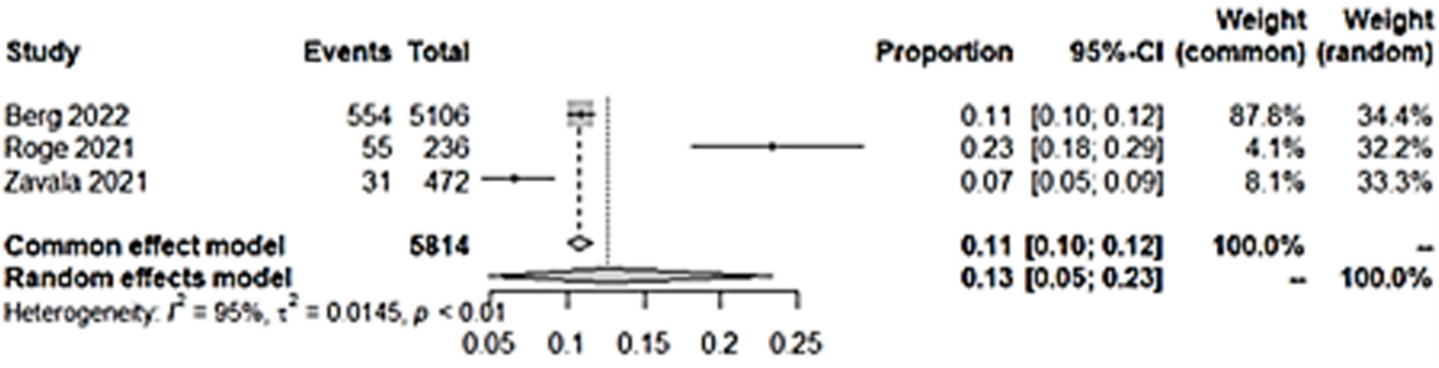

Fig 11. Pooled prevalence of mood swings in long COVID among children.

https://doi.org/10.1371/journal.pone.0282538.g011

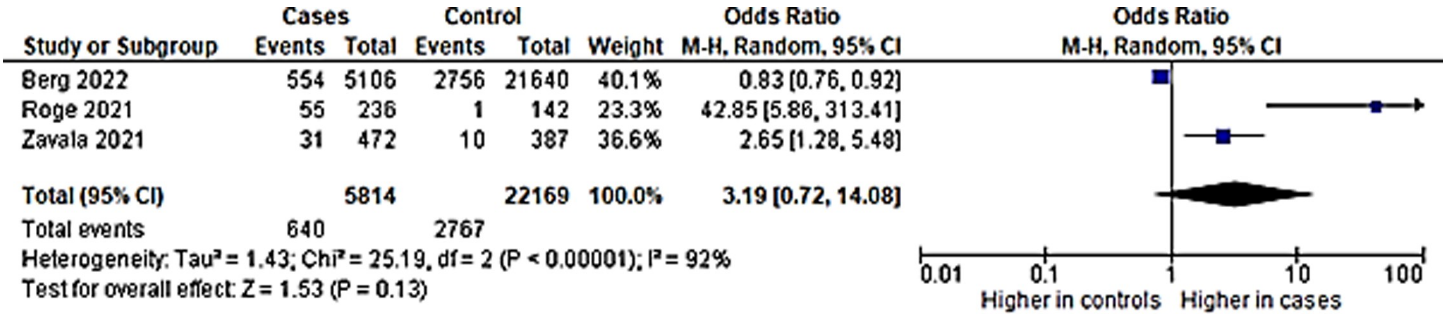

Fig 12. Mood swings in in long COVID among children with previous COVID-19 infection versus controls.

https://doi.org/10.1371/journal.pone.0282538.g012

CDI-S, and also had a high proportion of depression among controls at 32.9%, surpassing depression rate among cases in studies which assessed depression by a single symptom presence questions, at 4% [38] and 5.5% [37] (S1 Table). The study by Sterky and team had involved hospitalized children, but the children were hospitalized for COVID-19 between 13th March 2021 to 31st August 2021, which was about a year later than the study in Wuhan [37]. The proportion was much lower than the Wuhan study, despite only including children which were hospitalized due to COVID-19. Yet, this proportion was higher than the study by Zavala and team which involved children who were tested positive for COVID-19 regardless of hospitalization status. This may signify that hospitalized children may have a more severe condition of COVID-19 leading to higher anxiety and depression. However, for the study in Wuhan by Zhang and team, although the children were hospitalized, they were asymptomatic or had mild COVID-19 [35]. The decision to monitor these patients in the hospital earlier on in the pandemic may be deemed necessary by certain health authorities during early pandemic.

The event of being hospitalized and isolated may also be a contributing factor to develop mental health problems in hospitalized patients. Abrupt changes to a child's environment or routines, and separation from their social network were apparent in these cases. Quarantined children were found to be more prone to have psychological problems than children not undergoing quarantine [43]. Although this was partly due to COVID-19 worries and feelings of helplessness and fear, being isolated with changes in routines also might be a stressor to the child. They also have a higher possibility to have other family members being infected and affected from the virus. These psychological stressors and stressors due to the COVID-19 infection itself contributes to a prolonged stress in these children. Exposure to a severe or

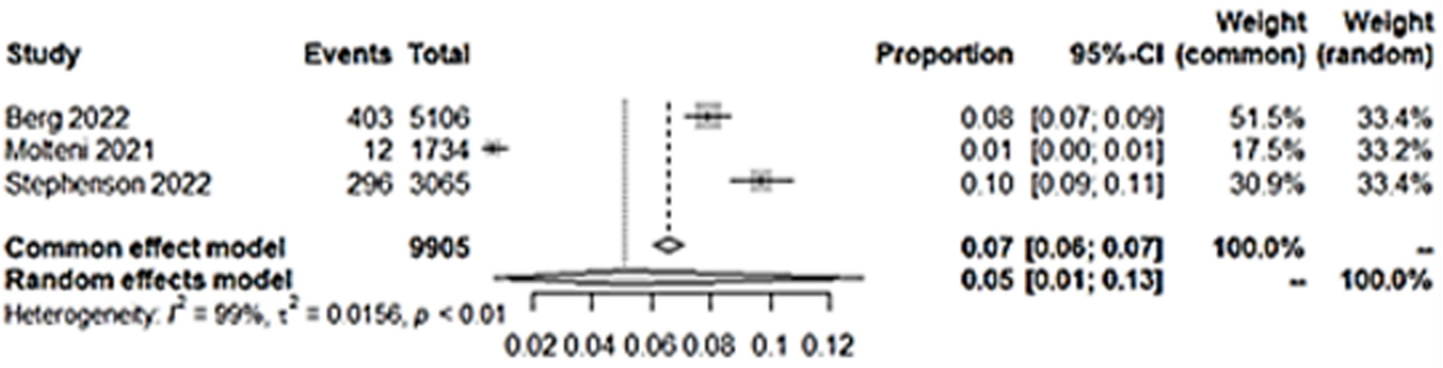

Fig 13. Pooled prevalence for appetite loss in long COVID among children.

https://doi.org/10.1371/journal.pone.0282538.g013

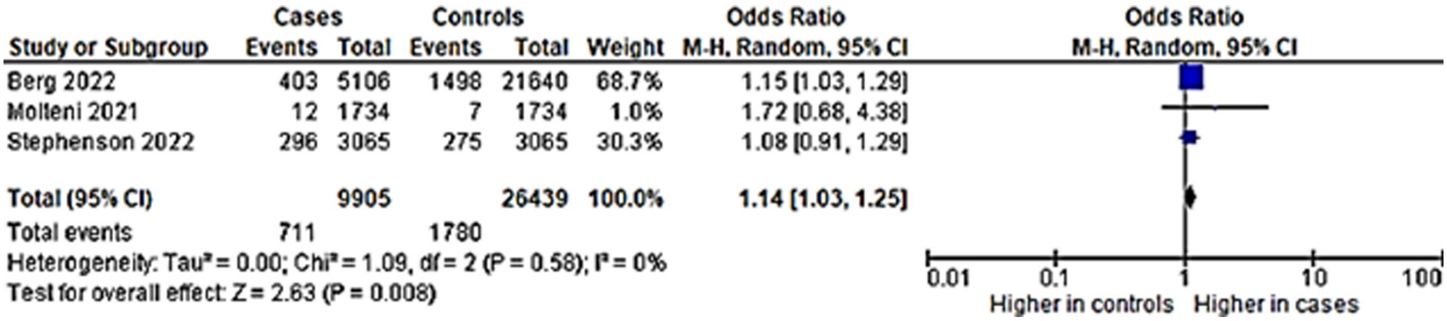

Fig 14. Appetite loss in long COVID among children with previous COVID-19 infection versus controls.

https://doi.org/10.1371/journal.pone.0282538.g014

prolonged stressor has long been shown to affect the body's physiological system and increase cortisol release. A prolonged activation of this system causes impairment in glucocorticoid receptors of the body, reduces the body's capacity to control inflammation and exacerbate production of pro-inflammatory cytokines [44]. The impact of inflammation on symptoms such as disturbed sleep and depression has been well documented, although the exact connecting mechanism between them was unable to be delineated [45]. Indeed, individuals with depression have been found to have higher levels of cortisol and pro-inflammatory cytokines, but this depends on both the severity and stage of the depression [46]. Despite this, in a study following-up patients one to four months after diagnosis of COVID-19, it was found that although the patients had a higher score on perceived stress, these score were not found to moderate the effects on mood or cognitive problems, possibly countering the theory that stress due to any cause were giving rise to the mood and cognitive problems [42].

Moreover, overall, the children with previous COVID-19, hospitalized and non-hospitalized, had more than two times higher odds of developing both depression and anxiety, compared to those without a previous COVID-19 infection. Therefore, it is logical that there is a contributing factor from the infection itself. Physical factors or symptoms may have contributed to the development of anxiety or depression, as it is not uncommon for people with long COVID to have multiple symptoms which involve both mental and physical health. The study by Stephenson and team showed that for children having previous COVID-19, at three

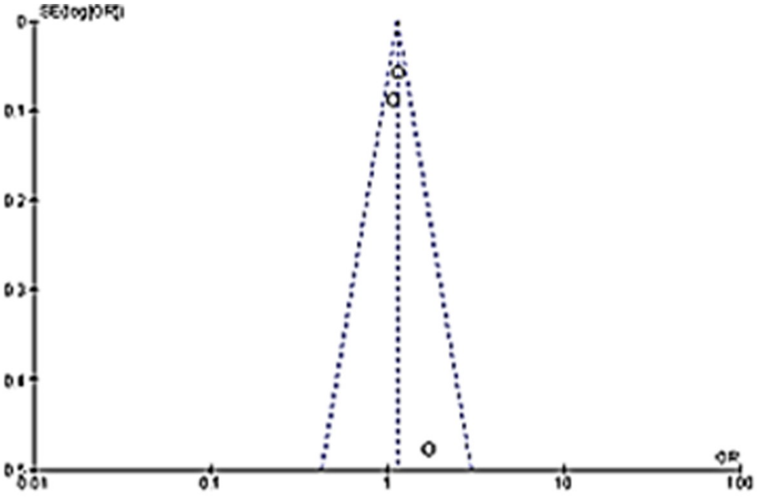

Fig 15. Funnel plot suggesting the heterogeneity among studies were related to sampling differences.

https://doi.org/10.1371/journal.pone.0282538.g015

months, aside from 66.5% still having symptoms, 30.3% had three or more symptoms [33]. Other studies also have shown that physical symptoms were associated with presence of the mental health symptoms. A study in Milan which followed-up COVID-19 patients for one to three months found that patients with physical symptoms of long COVID had 4.5 times higher odds for having anxiety or depression [47]. A similar finding was found in a study in Bangladesh, where persistent physical symptoms post-COVID-19 were significantly associated with depression among the long COVID patients [48]. However, this association was not only found in long COVID patients alone. A cohort study comparing RT-PCR positive and RT-PCR negative 11 to 17 years old children showed that those affected with multiple physical symptoms in both groups had poorer mental health, highlighting the reciprocal relationship between physical and mental health [33]. This relationship underscores the need for a robust assessment in those with long COVID, to assess both physical and psychological health symptoms which will contribute towards optimal management of the condition.

The association of physical symptoms with mental health symptoms signals that there might be underlying pathogenesis for both categories of symptoms, aside from compounding effects to and from each other. Some case reports did suggest organ damage in children post-COVID-19 similar to adults, which showed underlying pathogenesis harming multiple organs. A case report by Buonsenso and team showed that there were small vessels damage causing pulmonary dysfunction in an adolescent with post-acute sequela of COVID-19 [49]. The pathophysiology of this consequence was attributed to autoimmunity, inflammation or dysfunction of the inner lining of the blood vessels [50]. This pathogenesis affecting the nervous system might be one of the causes of the emergence of mental health problems. Studies have shown that the SARS-CoV-2 to be present in the cerebrovascular fluid in patients with COVID-19 [51], and affects the patient's brain findings in autopsies [52], providing evidence of the neurotropic effect and involvement in COVID-19 infection. Indeed, systemic inflammation has been attributed to cause neuropsychological and mental health symptoms such as depression, anxiety and cognitive defects [53]. In a prospective cohort study among adults, systemic inflammation scores predicted depression and cognitive impairment at three months, which signifies that the inflammation caused by COVID-19, predisposes patients to depression and cognitive problems [54].

This systematic inflammation or cytokine production in long COVID have also been implicated in other mental health symptoms such as loss of appetite. Our results support this, showing that a significant number of children with previous COVID-19 had appetite problems, with increased odds of 14% (OR:1.14, CI:1.03,1.25, $p = 0.008$), compared to those without. Thus, it suggests that the underlying pathophysiology of long COVID have an effect on appetite due to the underlying systematic inflammation, such as cytokine production. Regulation of appetite mechanism is suggested to be influenced by cytokines such as impairment of appetite in depression [55]. It may well be that in the three studies included (S1 Table), the appetite problems were related to depression, which were not assessed in all three studies. However, it may also be due to other problems such as underlying residual pathology from the COVID-19 infection, eating disorders, or any underlying physical problem such as a chronic infection. This was countered by using a control group to compare the incidence. The studies that assessed depression did not assess appetite problems as an outcome. Thus, the result on appetite problems is an analysis on its own. This underscores the need to assess appetite in children with a previous COVID-19 infection. The assessment and early intervention is very critical, as children need an optimal nutritional intake due to being in a critical period of neurodevelopment and growth [56]. Prolonged nutrition problems will likely lead to other future health morbidities in the affected children.

The results of our systematic review also showed that the mental health symptoms were high in studies which evaluated them from one month to three or four months (S1 Table). Therefore, we would recommend this time frame for screening and follow-up, which also coincided with the recommendation from the 'Italian Intersociety Consensus on Management of Long COVID in children'. The consensus recommends evaluation of the long COVID symptoms between 4 to 12 weeks, with an assessment at 4 weeks and at 12 weeks, and those with mental distress followed up appropriately using local resources for the problems [14]. On the other hand, NICE has not updated their guideline on follow-up and rehabilitation of long COVID-19 patients [11]. The recommendation for anxiety in NICE guidelines is to assess for other causes that may be reversible by exploring the person's anxiety and concerns and enlightening the carers on the issue and how to help, giving resources such as 'NHS every mind matters' and the 'Royal College of Paediatrics and Child Health resources for parents and carers' [11].

This systematic review also showed that screening using a validated questionnaire for each mental health problem would likely yield a higher proportion of children with problems. Therefore, we recommend using the validated questionnaire by healthcare services whenever the condition permits. This would ensure that children needing further support for mental health problems such as anxiety and depression acquire them, preventing mental health problems escalation and complications. NICE suggest using a questionnaire listing the most common symptoms in long COVID among children for evaluation during the initial assessment [11]. However, this should be followed up according to the clinical relevance and symptoms [14]. Multiple physical symptoms also should alert medical professionals to screen further for mental health symptoms [33].

It is clear from the systematic review that studies are lacking in the lower and middle-income countries regarding long COVID in children and mental health. It may well be that these countries had to focus on battling the pandemic and logically would need to focus more on the socio-economic impacts of the pandemic. Without sufficient data from these countries, the question on whether the decisions and recommendations pertaining to mental health in this group sufficiently apply and are appropriate to these countries remains. Therefore, the global community needs more advocacy regarding research and funding involving these countries.

There were a substantial number of studies on long COVID in children reviewed in the full-text phase, which did not assess the mental health states of the children with long COVID. In the few studies that evaluated mental health problems, only two studies used validated screening questionnaires specifically to assess anxiety and depression. One study was conducted in Wuhan, China which used SCARED, SDSC and CDI-S to assess anxiety, sleep problems and depression, respectively.

The main limitation of this meta-analysis is the number of studies included in each meta-analysis, which is due to the limited existing studies assessing mental health symptoms. Although a small number of studies may have caused the I^2 to be biased, it is still a valid measure along with the significance measured [57]. There were also limitations in the data collected, as some papers which contained information regarding children 18 years old but data could not be obtained from the authors. For example, there was one study from the United States with data on anxiety on long COVID among population less than 21 years old. However, feedback was not received from the corresponding author upon sent enquiring on the data via email.

One of the limitations of the analysis was using odds ratio instead of risk difference, which is more comprehensible to many. However, the metric of choice for this type of meta-analyses is shown to not be affecting the significance and only influenced the percentage identified

between-study heterogeneity and the results of Egger's test [58]. Egger's test was not done as data from proportional studies do not adjust adequately for this test [21].

Although this paper focused on the mental health problems in long COVID in children, the findings also highlighted the increased need to scale-up mental health screening and services in the general children population. This is evident from the results showing substantial proportions of mental health problems in the control population, despite being overall lower in anxiety and depression (Table 1). In the study in Wuhan, China for example, about 33% of the control group had depression and 23% had anxiety [35]. This emphasizes the need for a general measure and crisis plan, other than the focus on screening and intervention of anxiety and depression among long COVID in children.

Conclusion

The systematic review and meta-analysis of mental health problems in long COVID among children showed that anxiety and depression were significant mental health problems in the population. Children with previous COVID-19 infection had more than two times higher odds of having anxiety or depression and 14% higher odds of having appetite problems. The pooled prevalence of mental health problems among the population 5 to 15%. However, studies were heterogenous, and data are lacking from lower to middle-income countries (LMIC). The underlying causes for the depression, anxiety and appetite problems are potentially multifactorial, including caused by stressors, physical symptoms and systemic inflammation. The findings underscored the importance of screening and early intervention for children post-COVID-19 infection at one month and between three to four months. A scale-up of mental health screening and services is appropriate in children and adolescents, given the increased anxiety and depression, regardless of the status of previous COVID-19 infection. Proper screening using validated questionnaires for each mental health problem is needed for optimal detection, thus leading to a timely intervention, preventing future morbidity from the condition. Future research should use standardized tools and assessment time, and research from LMIC supported.

Supporting information

S1 Table. Summary of studies assessing each mental health outcomes with pooled prevalence results.
(DOCX)

S2 Table. Example of risk of bias/ quality assessment.
(DOCX)

S1 Checklist.
(TIF)

Acknowledgments

The authors would like to thank authors of the included studies who responded to email queries, if any.

Author Contributions

Conceptualization: Nurulhuda Mat Hassan, Hani Syahida Salim, Safiya Amaran, Norwati Daud, Deborah Fry.

Data curation: Nurulhuda Mat Hassan, Hani Syahida Salim, Safiya Amaran, Nurul Izza Yunus, Nurul Azreen Yusof, Norwati Daud.

Formal analysis: Nurulhuda Mat Hassan, Hani Syahida Salim, Safiya Amaran, Nurul Izza Yunus, Nurul Azreen Yusof, Norwati Daud, Deborah Fry.

Investigation: Nurulhuda Mat Hassan, Hani Syahida Salim.

Methodology: Nurulhuda Mat Hassan, Hani Syahida Salim, Safiya Amaran, Nurul Izza Yunus, Nurul Azreen Yusof, Norwati Daud, Deborah Fry.

Project administration: Nurulhuda Mat Hassan, Hani Syahida Salim, Safiya Amaran, Nurul Izza Yunus, Norwati Daud, Deborah Fry.

Resources: Nurulhuda Mat Hassan, Deborah Fry.

Software: Nurulhuda Mat Hassan, Deborah Fry.

Supervision: Deborah Fry.

Validation: Nurulhuda Mat Hassan, Hani Syahida Salim, Safiya Amaran, Nurul Izza Yunus, Nurul Azreen Yusof, Norwati Daud, Deborah Fry.

Visualization: Nurulhuda Mat Hassan, Hani Syahida Salim, Nurul Izza Yunus, Nurul Azreen Yusof, Deborah Fry.

Writing – original draft: Nurulhuda Mat Hassan, Hani Syahida Salim, Safiya Amaran, Nurul Izza Yunus, Nurul Azreen Yusof, Norwati Daud, Deborah Fry.

Writing – review & editing: Nurulhuda Mat Hassan, Hani Syahida Salim, Safiya Amaran, Nurul Azreen Yusof, Norwati Daud, Deborah Fry.

References

1. WHO. WHO Coronavirus (COVID-19) Dashboard: World Health Organization; 2022 [updated 2022; cited 2022 September]. Available from: https://covid19.who.int/.
2. Patel V, Saxena S, Lund C, Thornicroft G, Baingana F, Bolton P, et al. The Lancet Commission on global mental health and sustainable development. The Lancet. 2018; 392(10157):1553–98. https://doi.org/10.1016/S0140-6736(18)31612-X PMID: 30314863
3. Guan M. Could the associations of changes in living arrangement with mental disorders be moderated or mediated during covid-19 pandemic? Psychology Research and Behavior Management. 2021; 14:769–79. https://doi.org/10.2147/PRBM.S302729 PMID: 34168508
4. WHO. WHO Director-General's opening remarks at the media briefing on COVID-19–11 March 20202020. Available from: https://www.who.int/director-general/speeches/detail/who-director-general-s-opening-remarks-at-the-media-briefing-on-covid-19—11-march-2020.
5. Racine N, McArthur BA, Cooke JE, Eirich R, Zhu J, Madigan S. Global Prevalence of Depressive and Anxiety Symptoms in Children and Adolescents During COVID-19: A Meta-analysis. JAMA Pediatrics. 2021; 175(11):1142–50. https://doi.org/10.1001/jamapediatrics.2021.2482 PMID: 34369987
6. Polanczyk GV, Salum GA, Sugaya LS, Caye A, Rohde LA. Annual Research Review: A meta-analysis of the worldwide prevalence of mental disorders in children and adolescents. Journal of Child Psychology and Psychiatry. 2015; 56(3):345–65. https://doi.org/10.1111/jcpp.12381 PMID: 25649325
7. Lu W. Adolescent Depression: National Trends, Risk Factors, and Healthcare Disparities. American Journal of Health Behavior. 2019; 43(1):181–94. https://doi.org/10.5993/AJHB.43.1.15 PMID: 30522576
8. Simpson F, Chew-Graham C, Lokugamage A. Life & times long COVID in children: The perspectives of parents and children need to be heard. British Journal of General Practice. 2021; 71(706):216.
9. Aiyegbusi OL, Hughes SE, Turner G, Rivera SC, McMullan C, Chandan JS, et al. Symptoms, complications and management of long COVID: a review. Journal of the Royal Society of Medicine. 2021; 114 (9):428–42. https://doi.org/10.1177/01410768211032850 PMID: 34265229
10. CDC. Post-COVID Conditions: Overview for Healthcare Providers 2021. Available from: https://www.cdc.gov/coronavirus/2019-ncov/hcp/clinical-care/post-covid-conditions.html.

11. NICE. COVID-19 rapid guideline: managing the long term effects of COVID-192021. Available from: www.nice.org.uk/guidance/ng188.

12. WHO. A clinical case definition of post COVID-19 condition by a Delphi consensus, 6 October 2021. Clinical Services and Systems, Communicable Diseases, Technical Advisory Group on SARS-CoV-2 Virus Evolution; 2021.

13. Berg SK, Palm P, Nielsen SD, Nygaard U, Bundgaard H, Rotvig C, et al. Acute symptoms in SARS-CoV-2 positive adolescents aged 15–18 years–Results from a Danish national cross-sectional survey study. The Lancet Regional Health—Europe. 2022; 16. https://doi.org/10.1016/j.lanepe.2022.100354 PMID: 35350632

14. Esposito S, Principi N, Azzari C, Cardinale F, Di Mauro G, Galli L, et al. Italian intersociety consensus on management of long covid in children. Italian Journal of Pediatrics. 2022; 48(1). https://doi.org/10.1186/s13052-022-01233-6 PMID: 35264214

15. Behnood SA, Shafran R, Bennett SD, Zhang AXD, O'Mahoney LL, Stephenson TJ, et al. Persistent symptoms following SARS-CoV-2 infection amongst children and young people: A meta-analysis of controlled and uncontrolled studies. JOURNAL OF INFECTION. 2022; 84(2):158–70. https://doi.org/10.1016/j.jinf.2021.11.011 PMID: 34813820

16. Schou TM, Joca S, Wegener G, Bay-Richter C. Psychiatric and neuropsychiatric sequelae of COVID-19-A systematic review. Brain Behavior and Immunity. 2021; 97:328–48. https://doi.org/10.1016/j.bbi.2021.07.018 PMID: 34339806

17. Ashkenazi-Hoffnung L, Shmueli E, Ehrlich S, Ziv A, Bar-On O, Birk E, et al. Long COVID in Children: Observations From a Designated Pediatric Clinic. The Pediatric Infectious Disease Journal. 2021; 40 (12). https://doi.org/10.1097/INF.0000000000003285 PMID: 34371507

18. Buonsenso D, Pujol FE, Munblit D, Pata D, McFarland S, Simpson FK. Clinical characteristics, activity levels and mental health problems in children with long coronavirus disease: a survey of 510 children. Future Microbiology. 2022; 17(8):577–88. https://doi.org/10.2217/fmb-2021-0285 PMID: 35360923

19. Clavenna A, Di Francesco C, Di Maio L, Mauri L, Narducci M, Schirò R, et al. Risk of Sequelae of COVID-19 in Children Cared for by Primary Care Pediatricians. Indian Pediatrics. 2022; 59(1):87–8. https://doi.org/10.1007/s13312-022-2427-3 PMID: 34845989

20. Buonsenso D, Gennaro LD, Rose CD, Morello R, D'Ilario F, Zampino G, et al. Long-Term outcomes of pediatric infections: From traditional infectious diseases to long Covid. Future Microbiology. 2022; 17 (7):551–71.

21. Barker TH, Migliavaca CB, Stein C, Colpani V, Falavigna M, Aromataris E, et al. Conducting proportional meta-analysis in different types of systematic reviews: a guide for synthesisers of evidence. BMC Medical Research Methodology. 2021; 21(1):189. https://doi.org/10.1186/s12874-021-01381-z PMID: 34544368

22. UNCHR. Convention on the Rights of the Child text 2021 [Available from: https://www.unicef.org/child-rights-convention/convention-text-childrens-version.

23. Fletcher J. What is heterogeneity and is it important? BMJ. 2007; 334(7584):94. https://doi.org/10.1136/bmj.39057.406644.68 PMID: 17218716

24. Sedgwick P. Meta-analyses: tests of heterogeneity. BMJ: British Medical Journal. 2012; 344:e3971.

25. Sterne JAC, Sutton AJ, Ioannidis JPA, Terrin N, Jones DR, Lau J, et al. Recommendations for examining and interpreting funnel plot asymmetry in meta-analyses of randomised controlled trials. BMJ. 2011; 343:d4002. https://doi.org/10.1136/bmj.d4002 PMID: 21784880

26. Cumming G. Understanding the new statistics. New York: Taylor and Francis Group; 2012.

27. Kim HY. Statistical notes for clinical researchers: Risk difference, risk ratio, and odds ratio. Restor Dent Endod. 2017; 42(1):72–6. https://doi.org/10.5395/rde.2017.42.1.72 PMID: 28194368

28. Page MJ, McKenzie JE, Bossuyt PM, Boutron I, Hoffmann TC, Mulrow CD, et al. The PRISMA 2020 statement: an updated guideline for reporting systematic reviews. BMJ. 2021; 372:n71. https://doi.org/10.1136/bmj.n71 PMID: 33782057

29. Lee SW, Koo MJ. PRISMA 2020 statement and guidelines for systematic review and meta-analysis articles, and their underlying mathematics: Life Cycle Committee Recommendations. Life Cycle. 2022; 2: e9.

30. Blankenburg J, Wekenborg MK, Reichert J, Kirsten C, Kahre E, Haag L, et al. Comparison of mental health outcomes in seropositive and seronegative adolescents during the COVID19 pandemic. Scientific Reports. 2022; 12(1). https://doi.org/10.1038/s41598-022-06166-y PMID: 35145161

31. Radtke T, Ulyte A, Puhan M, Kriemler A. Long-term symptoms after SARS-CoV-2 infection in school children: population-based cohort with 6-months follow-up. medRxiv. 2021.

32. Allen N, Brady M, Ni Riain U, Conlon N, Domegan L, Carrion Martin AI, et al. Prevalence of Antibodies to SARS-CoV-2 Following Natural Infection and Vaccination in Irish Hospital Healthcare Workers:

Changing Epidemiology as the Pandemic Progresses. Frontiers in Medicine. 2022; 8. https://doi.org/10.3389/fmed.2021.758118 PMID: 35186963

33. Stephenson T, Shafran R, De Stavola B, Rojas N, Aiano F, Amin-Chowdhury Z, et al. Long COVID and the mental and physical health of children and young people: National matched cohort study protocol (the CLoCk study). BMJ Open. 2021; 11(8). https://doi.org/10.1136/bmjopen-2021-052838 PMID: 34446502

34. Roge I, Smane L, Kivite-Urtane A, Pucuka Z, Racko I, Klavina L, et al. Comparison of Persistent Symptoms After COVID-19 and Other Non-SARS-CoV-2 Infections in Children. Frontiers in Pediatrics. 2021; 9:752385. https://doi.org/10.3389/fped.2021.752385 PMID: 34778143

35. Zhang XB, Yang HD, Zhang J, Yang M, Yuan NA, Liu JJ. Prevalence of and risk factors for depressive and anxiety symptoms in a large sample of Chinese adolescents in the post-COVID-19 era. CHILD AND ADOLESCENT PSYCHIATRY AND MENTAL HEALTH. 2021; 15(1).

36. Osmanov IM, Spiridonova E, Bobkova P, Gamirova A, Shikhaleva A, Andreeva M, et al. Risk factors for long covid in previously hospitalised children using the ISARIC Global follow-up protocol: A prospective cohort study. European Respiratory Journal. 2022; 59(2).

37. Sterky E, Olsson-Åkefeldt S, Hertting O, Herlenius E, Alfven T, Ryd Rinder M, et al. Persistent symptoms in Swedish children after hospitalisation due to COVID-19. Acta Paediatrica. 2021; 110(9):2578–80. https://doi.org/10.1111/apa.15999 PMID: 34157167

38. Zavala M, Ireland G, Amin-Chowdhury Z, Ramsay ME, Ladhani SN. Acute and Persistent Symptoms in Children With Polymerase Chain Reaction (PCR)-Confirmed Severe Acute Respiratory Syndrome Coronavirus 2 (SARS-CoV-2) Infection Compared With Test-Negative Children in England: Active, Prospective, National Surveillance. Clinical Infectious Disease. 2021.

39. FDA. Antibody (Serology) Testing for COVID-19: Information for Patients and Consumers: U.S. Food and Drug Administration; 2022 [updated 24 February 2022. Available from: https://www.fda.gov/medical-devices/coronavirus-covid-19-and-medical-devices/antibody-serology-testing-covid-19-information-patients-and-consumers.

40. Allen JG, Romate J, Rajkumar E. Mindfulness-based positive psychology interventions: a systematic review. BMC Psychology. 2021; 9(1). https://doi.org/10.1186/s40359-021-00618-2 PMID: 34362457

41. Gong F, Wei H-x, Li Q, Liu L, Li B. Evaluation and Comparison of Serological Methods for COVID-19 Diagnosis. Frontiers in Molecular Biosciences. 2021; 8. https://doi.org/10.3389/fmolb.2021.682405 PMID: 34368226

42. Lamontagne SJ, Winters MF, Pizzagalli DA, Olmstead MC. Post-acute sequelae of COVID-19: Evidence of mood & cognitive impairment. Brain Behav Immun Health. 2021; 17:100347.

43. Saurabh K, Ranjan S. Compliance and Psychological Impact of Quarantine in Children and Adolescents due to Covid-19 Pandemic. The Indian Journal of Pediatrics. 2020; 87(7):532–6. https://doi.org/10.1007/s12098-020-03347-3 PMID: 32472347

44. Nandam LS, Brazel M, Zhou M, Jhaveri DJ. Cortisol and Major Depressive Disorder-Translating Findings From Humans to Animal Models and Back. Front Psychiatry. 2019; 10:974. https://doi.org/10.3389/fpsyt.2019.00974 PMID: 32038323

45. Bouças AP, Rheinheimer J, Lagopoulos J. Why Severe COVID-19 Patients Are at Greater Risk of Developing Depression: A Molecular Perspective. The Neuroscientist. 2020; 28(1):11–9. https://doi.org/10.1177/1073858420967892 PMID: 33135582

46. Stetler C, Miller GE. Depression and hypothalamic-pituitary-adrenal activation: a quantitative summary of four decades of research. Psychosom Med. 2011; 73(2):114–26. https://doi.org/10.1097/PSY.0b013e31820ad12b PMID: 21257974

47. Tomasoni D, Bai F, Castoldi R, Barbanotti D, Falcinella C, Mulè G, et al. Anxiety and depression symptoms after virological clearance of COVID-19: A cross-sectional study in Milan, Italy. J Med Virol. 2021; 93(2):1175–9. https://doi.org/10.1002/jmv.26459 PMID: 32841387

48. Islam MS, Ferdous MZ, Islam US, Mosaddek ASM, Potenza MN, Pardhan S. Treatment, persistent symptoms, and depression in people infected with covid-19 in bangladesh. International Journal of Environmental Research and Public Health. 2021; 18(4):1–16. https://doi.org/10.3390/ijerph18041453 PMID: 33562427

49. Buonsenso D, Di Giuda D, Sigfrid L, Pizzuto DA, Di Sante G, De Rose C, et al. Evidence of lung perfusion defects and ongoing inflammation in an adolescent with post-acute sequelae of SARS-CoV-2 infection. The Lancet Child and Adolescent Health. 2021; 5(9):677–80. https://doi.org/10.1016/S2352-4642(21)00196-6 PMID: 34339624

50. Nalbandian A, Sehgal K, Gupta A, Madhavan MV, McGroder C, Stevens JS, et al. Post-acute COVID-19 syndrome. Nature Medicine. 2021; 27(4):601–15. https://doi.org/10.1038/s41591-021-01283-z PMID: 33753937

51. Zhou Z, Kang H, Li S, Zhao X. Understanding the neurotropic characteristics of SARS-CoV-2: from neurological manifestations of COVID-19 to potential neurotropic mechanisms. J Neurol. 2020; 267 (8):2179–84. https://doi.org/10.1007/s00415-020-09929-7 PMID: 32458193

52. Reichard RR, Kashani KB, Boire NA, Constantopoulos E, Guo Y, Lucchinetti CF. Neuropathology of COVID-19: a spectrum of vascular and acute disseminated encephalomyelitis (ADEM)-like pathology. Acta Neuropathologica. 2020; 140(1):1–6. https://doi.org/10.1007/s00401-020-02166-2 PMID: 32449057

53. Rhoades R, Solomon S, Johnson C, Teng S. Impact of SARS-CoV-2 on Host Factors Involved in Mental Disorders. Frontiers in Microbiology. 2022; 13. https://doi.org/10.3389/fmicb.2022.845559 PMID: 35444632

54. Mazza MG, Palladini M, De Lorenzo R, Magnaghi C, Poletti S, Furlan R, et al. Persistent psychopathology and neurocognitive impairment in COVID-19 survivors: Effect of inflammatory biomarkers at three-month follow-up. Brain Behav Immun. 2021; 94:138–47. https://doi.org/10.1016/j.bbi.2021.02.021 PMID: 33639239

55. Andréasson A, Arborelius L, Erlanson-Albertsson C, Lekander M. A putative role for cytokines in the impaired appetite in depression. Brain Behav Immun. 2007; 21(2):147–52. https://doi.org/10.1016/j.bbi.2006.08.002 PMID: 16996241

56. Yang HR. How to approach feeding difficulties in young children. Korean J Pediatr. 2017; 60(12):379–84. https://doi.org/10.3345/kjp.2017.60.12.379 PMID: 29302261

57. Von Hippel PT. The heterogeneity statistic I(2) can be biased in small meta-analyses. BMC Med Res Methodol. 2015; 15:35. https://doi.org/10.1186/s12874-015-0024-z PMID: 25880989

58. Papageorgiou SN, Tsiranidou E, Antonoglou GN, Deschner J, Jäger A. Choice of effect measure for meta-analyses of dichotomous outcomes influenced the identified heterogeneity and direction of small-study effects. J Clin Epidemiol. 2015; 68(5):534–41. https://doi.org/10.1016/j.jclinepi.2015.01.004 PMID: 25666885
